# Categorizing 161 plant (streptophyte) mitochondrial group II introns into 29 families of related paralogues finds only limited links between intron mobility and intron-borne maturases

**DOI:** 10.1186/s12862-023-02108-y

**Published:** 2023-03-13

**Authors:** Simon Zumkeller, Volker Knoop

**Affiliations:** IZMB, Institut für Zelluläre und Molekulare Botanik, Abteilung Molekulare Evolution, Universität Bonn, Kirschallee 1, 53115 Bonn, Germany

**Keywords:** Group II introns, Retrotransposition, Maturases, Group I introns, mtDNA, Mitogenomes, Horizontal gene transfer

## Abstract

Group II introns are common in the two endosymbiotic organelle genomes of the plant lineage. Chloroplasts harbor 22 positionally conserved group II introns whereas their occurrence in land plant (embryophyte) mitogenomes is highly variable and specific for the seven major clades: liverworts, mosses, hornworts, lycophytes, ferns, gymnosperms and flowering plants. Each plant group features “signature selections” of ca. 20–30 paralogues from a superset of altogether 105 group II introns meantime identified in embryophyte mtDNAs, suggesting massive intron gains and losses along the backbone of plant phylogeny. We report on systematically categorizing plant mitochondrial group II introns into “families”, comprising evidently related paralogues at different insertion sites, which may even be more similar than their respective orthologues in phylogenetically distant taxa. Including streptophyte (charophyte) algae extends our sampling to 161 and we sort 104 streptophyte mitochondrial group II introns into 25 core families of related paralogues evidently arising from retrotransposition events. Adding to discoveries of only recently created intron paralogues, hypermobile introns and twintrons, our survey led to further discoveries including previously overlooked “fossil” introns in spacer regions or e.g., in the *rps8* pseudogene of lycophytes. Initially excluding intron-borne maturase sequences for family categorization, we added an independent analysis of maturase phylogenies and find a surprising incongruence between intron mobility and the presence of intron-borne maturases. Intriguingly, however, we find that several examples of nuclear splicing factors meantime characterized simultaneously facilitate splicing of independent paralogues now placed into the same intron families. Altogether this suggests that plant group II intron mobility, in contrast to their bacterial counterparts, is not intimately linked to intron-encoded maturases.

**Supplementary Information:**

The online version contains supplementary material available at 10.1186/s12862-023-02108-y.

## Introduction

**Fig. 1:**
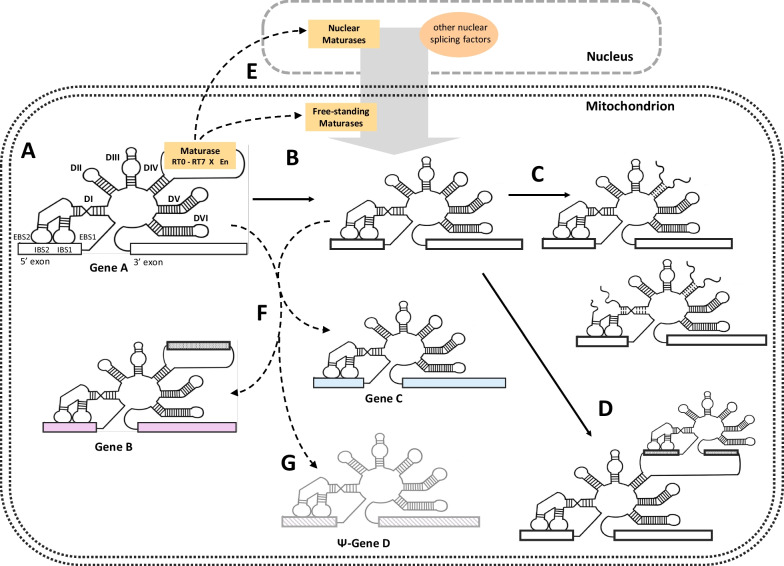
Plant mitochondrial group II introns evolving. **A** In its likely evolutionary ancestral state, a group II intron encodes a multifunctional maturase in domain IV of its characteristic six-domain structure (DI through DVI). A fully equipped maturase features reverse transcriptase domains RT0-RT7 (the finger and palm domains), followed by a maturase-specific ‘X’ domain and a DNA endonuclease ‘En’ domain with relevance for intron mobility. Interaction of exon binding sites (EBS 1 and 2) in domain DI with corresponding intron binding sites at the end of the upstream exon are equally important for splicing and retromobility. **B** Intron-borne maturases frequently degenerate and get lost during plant mitogenome evolution. **C** Multiple cases of *cis*-to-*trans*-splicing transitions are evident for plant mitochondrial group II introns, creating bipartite introns mostly broken in domain DIV or even tripartite group II introns [10]. **D** Plant mitochondrial group II introns may invade other introns, creating twintrons [14]. **E** The contribution of group II intron-encoded maturases, their free-standing paralogues in plant mitochondrial or nuclear genomes and those of multiple phylogenetically unrelated splicing factors [e.g. 19, 20, 47, 130] to all the evolutionary processes summarized in the figure is not fully understood at present. **F** Of particular relevance in this survey is the capability of plant mitochondrial group II introns to occupy new gene locations, creating sequence-related group II paralogues, here grouped into families. **G** The complete survey of plant mitochondrial group II introns presented includes paralogues that degenerated together with their host genes, or which can be identified as “fossil” introns in intergenic locations. Group II intron illustration have been made manually with ProCreate^®^

At first glance, the two endosymbiont genomes in the plant cell seem to have followed very similar evolutionary trajectories with massive gene transfers into the nucleus, strongly reducing the ancestral genomes of an α-proteobacterium and a cyanobacterium that gave rise to mitochondria and chloroplasts, respectively. Even later emerging molecular mechanisms such as the site-specific C-to-U RNA editing characteristic for land plants affect the transcriptomes of both plant organelles equally [1, 2]. In contrast, another characteristic feature of the two endosymbiont genomes in plants shows a striking discrepancy: the overwhelming stasis in the occurrence of group II introns in chloroplast DNAs for more than 500 million years, even extending into the streptophyte algal ancestors vs. the strikingly dynamic evolution of their counterparts in the mitochondrial genomes of streptophytes, i.e. land plants (embryophytes) and the related green algal lineages (charophytes) tracing to a common ancestor. Examples for the latter are documented with the multiple splicing pathways of plant mitochondrial group II introns [3–5], their many transitions from *cis*- into *trans*-splicing arrangements [e.g. 6–13], the recently discovered functional or degenerated twintrons [14] or the evident degeneration of intron-borne maturase reading frames [6, 15] and the creation of nuclear-encoded maturase genes [16–21] in the multifarious pathways of evolution (Fig. 1A–E). Here, we focus on the occupation of new loci by mobile plant mitochondrial group II introns, including examples of introns “fossilized” in pseudogenes or intergenic regions (Fig. 1F, G).

The peculiar clade-specific patterns in the occurrence of plant mitochondrial group II introns [22–24] and the examples for evolutionary recent occupations of new intron insertion sites [25, 26] suggest that plant mitogenomes could offer a particularly rich and attractive data source to elucidate the mobility of group II introns on a broad evolutionary scale. Previous research on group II intron mobility has so far largely focused on functional studies with selected group II model introns in fungi or bacteria that have helped to elucidate the biochemical pathways of intron propagation and invasion of new loci [27–29]. Moreover, many features make group II introns particularly interesting ribozyme RNAs in general [30–39]: Firstly, the biochemical mechanism of their splicing, involving two transesterifications using a 5′-terminal guanosine and a looped out adenosine upstream of the 3′-splice acceptor site, is homologous to that of spliceosomal introns in the nuclear genomes of eukaryotes. In fact, all available evidence convincingly suggests group II introns to be the evolutionary ancestors of the eukaryotic spliceosome machinery [40–43]. Secondly, some group II introns have been demonstrated to have self-splicing activity in vitro, a notable case being the second intron in the mitochondrial *rrnL* gene of the brown alga *Pylaiella littoralis* [44] that splices efficiently even at low Mg^2+^ ion concentrations. Thirdly, in their likely evolutionary ancestral state, group II introns carry reading frames for maturases, intron-encoded proteins (IEPs) that are crucial to assist both in splicing but also in the mobility, allowing their host introns to occupy new insertion sites (Fig. 1A). In their most complete forms maturases consist of DNA-binding and endonucleolytic cleavage (D/En) and reverse transcriptase (RT) domains allowing the conversion of the RNA spliced into a novel DNA location, thereby creating a new intron paralogue.

However, none of the particularly abundant group II introns in plant organelle genomes have been experimentally demonstrated to be mobile and reports on self-splicing or in vitro splicing in the presence of chloroplast extracts, respectively, have only recently been published [45, 46]. The lack of plant mitochondrial group II intron self-splicing activity is likely associated with the diverse set of nuclear-encoded proteins that act as either intron-specific or more promiscuous splicing factors [47, 48] and which continue to be characterized in ever-increasing numbers by reverse genetic studies, as it is easily documented with only some most recently published examples [49–53].

While studies of group II intron mobility in plant mitogenomes are hampered by lacking experimental approaches to manipulate plant mtDNAs, the extraordinary diversity of introns in plant mitogenomes offers an intriguing evolutionary perspective. Sequence similarities between plant mitochondrial intron paralogues had become evident soon after the first complete plant mitogenome sequences became available [54]. For example, the overall sequence similarity between the first intron in the *nad2* gene (nad2i156g2) and the second intron of the *nad1* gene (nad1i477g2) in flowering plant mitogenomes has been recognized early [55]. Similarly, the case of nad5i392g2 is striking, an intron that is phylogenetically very restricted to the family Lycopodiaceae among the lycophytes but clearly related to downstream intron nad5i1242g2 in the *nad5* gene, which has a much wider phylogenetic conservation among lycophytes and ferns [56]. More recently emerging examples for evidently related group II intron paralogue pairs are two cases in ferns: atp1i361g2 and rps3i249g2 [26] and rps1i25g2 and rpl2i846g2 [25]. The hitherto most striking example of extraordinary sequence similarity between two intron paralogues has only very recently been discovered in hornwort mtDNAs accompanying the discovery of twintrons [14]. Intron paralogue cox2i98g2 is exclusively present in the hornwort genus *Anthoceros* and shares more than 98% sequence similarity with the internal group II intron of a twintron in the *atp1* gene (atp1i1050g2ii1536g2), which is widely distributed among the hornworts. This example immediately suggests a recent copying event from *atp1* into *cox2* late in the recent phylogeny of hornworts.

Here, we systematically scanned available mitogenomes of streptophytes (i.e., land plants and the phylogenetically most closely related “charophyte” green algae) and scored the relationships between their group II introns as an effort to elucidate how intron migrations and losses may have contributed to shape the mitogenome makeups in extant plants. We define 25 core “families” F01–F25 of clearly sequence-related group II intron paralogues, which indicate ancient and recent intron copying events. With the extraordinary slow sequence evolution in mitogenomes of the plant lineage combined with an increasingly well-understood phylogeny of land plants and related streptophyte algae our approach will offer a new perspective on an evolutionary timescale covering more than 500 million years. Altogether, we were able to assign 104 from a total of 161 streptophyte mitochondrial group II introns to one of the now defined 25 families whereas the 57 remaining introns presently lack significant similarity to any other paralogues. We discuss possible intron migration scenarios, considering the likely important role of intron-encoded or free-standing maturases.

## Results

### The streptophyte mitochondrial group II intron data set

We scanned plant mitochondrial genomes for the occurrence of group II introns, including the complete phylogenetic diversity representing the seven major land plant clades: flowering plants (angiosperms), gymnosperms, ferns (Monilophytes), lycophytes, hornworts (Anthocerotophyta), mosses (Bryophyta) and liverworts (Marchantiophyta). We additionally included the available mitogenomes of streptophyte algae (“Charophytes”) representing six classes: Zygnematophyceae (Desmidiales: *Closterium bailyanum, Gonatozygon brebissonii*; Zygnematales: *Entransia fimbriata, Roya anglica, R. obtusa* and *Zygnema circumcarinatum*) recently considered to be most closely related to the plant lineage as well as Charophyceae (*Chara vulgaris, C. braunii, Nitella hyalina*), Coleochaetophyceae (*Chaetosphaeridium globosum, Coleochaete scutata*), Chlorokybophyceae (*Chlorokybus atmophyticus*), Klebsormidiophyceae (*Klebsormidium flaccidum*) and the early-branching Mesostigmatophyceae (*Mesostigma viride*). For a clear designation of introns we use the previously suggested intron nomenclature based on the intron insertion site in a given gene behind the reference position in the respective homologue of the liverwort *Marchantia polymorpha* [57, 58].

**Fig. 2:**
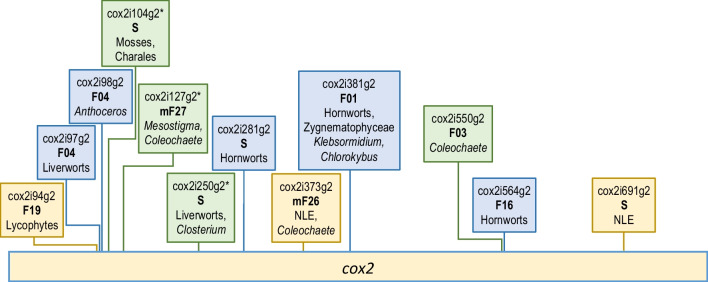
The *cox2* gene. The example of the *cox2* gene illustrates important issues of streptophyte mitochondrial group II intron diversity. Altogether twelve different group II insertion sites are presently identified in different plant lineages and streptophyte algae with colors distinguishing introns in bryophytes (blue), those shared with or present only in algae (green) and those shared with or present only in vascular plants (yellow). The latter include cox2i373g2 and cox2i691g labeled with NLE for “Non-Liverwort Embryophytes” given their presence in all land plant lineages except the liverworts. Asterisks indicate cases of introns occupying the same position in algae and land plants that were considered orthologues despite lack of significant sequence similarity

The example of the *cox2* gene (Fig. 2) is used to introduce into important issues of our analyses addressing the huge diversity of mitochondrial introns in the plant lineage. Altogether twelve different group II insertion sites are presently identified in the *cox2* genes of land plants and streptophyte algae. Their phylogenetic distributions vary widely from introns present in most land plant lineages excluding liverworts (cox2i373g2 and cox2i691g2) to others presently only identified in the hornwort genus *Anthoceros* (cox2i98g2) or in the streptophyte alga *Coleochaete scutata* (cox2i550g2). The assignments of introns to core families, which comprise significantly similar intron paralogues as introduced in this work, are given below the intron names: cox2i381g2 in family F01, cox2i550g2 in F03, cox2i97g2 and cox2i98g2 in F04, cox2i564g2 in F16 and cox2i94g2 in F19 (see below). Including intron-based maturases allows for definition of maturase-based families and superfamilies, here cox2i373g2 in mF26 and cox2i127g2 in mF27. Remaining “solitary” introns lacking significant similarity to other paralogues are labeled “S”. Insertion sites must be considered very carefully and precisely, notably when introns occur differently in a small gene region such as introns cox2i94g2, cox2i97g2, cox2i98g2 and cox2i104g2, an issue occasionally overlooked and leading to mis-annotation in database entries. Introns in the same position were considered as orthologues also when lacking significant sequence similarities across large phylogenetic distances if they were not assigned to different families.

Our re-evaluation of database entries during intron sampling has made us re-consider several intron and splice site annotations and allowed us to suggest yet further, and very likely functionally splicing, introns that were previously unnoticed but would reconstitute important and conserved parts of their host genes (e.g. cox3i34g2 in *Gonatozygon*, nad2i81g2 in *Zygnema* or nad9i89g2 in *Closterium* and *Gonatozygon*) as well as dysfunctional, “fossil” introns (e.g. rps8i52g2f in *Phlegmariurus*), as we will discuss below. Our final total group II intron sampling in streptophyte mitogenomes comprised 161 group II intron paralogues defined by their unique insertion sites.

**Fig. 3:**
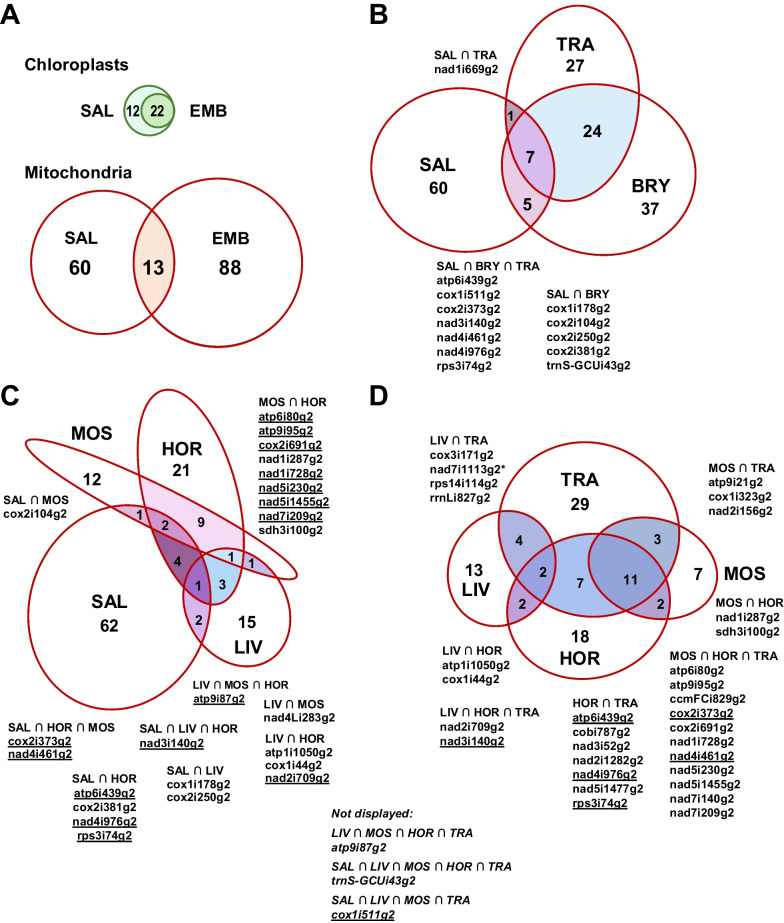
Euler diagrams of streptophyte mitochondrial group II intron distributions. Euler diagrams displaying the occurrence of organelle group II introns. **A** 22 chloroplast group II introns (green circles) of embryophytes (EMB) are a highly conserved subset of 34 homologues in streptophyte algae (SAL) whereas of 161 mitochondrial group II introns in streptophyte mitogenomes (red circles) only 13 are shared between streptophyte algae and land plants. **B** Mitochondrial group II intron paralogues present in the specific intersections are listed for Streptophyte algae (SAL), Tracheophytes (TRA) and Bryophytes (BRY) and, in **C** and **D**, more specifically for the three bryophyte clades of Liverworts (LIV), Mosses (MOS) and Hornworts (HOR). Underlined in **C** are introns also occurring in tracheophytes and, vice versa, in **D** those also present in streptophyte algae. An asterisk indicates that the status of intron nad7i1113g2 is unclear owing to pseudogene degeneration. Highlighted in red in panel C is intron atp9i87g2 as the only intron shared between the three bryophyte classes, which is not displayed in **D** owing to limitations of Euler displays. Likewise, intron cox1i511g2, highlighted below in italics, is not displayed in **C** and **D**

The occurrence of streptophyte mitochondrial group II intron paralogues in the major clades is displayed with Euler diagrams in Fig. 3. The striking discrepancy of mitochondrial vs. chloroplast group II introns is immediately apparent with 22 land plant chloroplast introns all of which have counterparts in streptophyte algae vs. altogether 161 mitochondrial group II introns, of which only 13 are shared between embryophytes and streptophyte algae (Fig. 3A). Differentiating among the embryophytes, larger intersections are found between bryophytes and tracheophytes (Fig. 3B) than with either group and the outgroup algae and among the latter between hornworts and mosses (Fig. 3C) and between hornworts, mosses and tracheophytes (Fig. 3D), respectively. Notably, of 101 group II introns identified in embryophyte mtDNAs, only one (atp9i87g2) is shared between all three bryophyte clades (Fig. 3C).

### The group II intron family concept

Despite their highly conserved six-domain structures, group II intron paralogues a priori share no significant sequence similarities aside from mostly conserved sequence motifs at the 5′-end (mostly GUGCG), the 3′- termini (mostly AY) and their characteristic domain V comprising 34 nucleotides, which mostly folds into two base-paired regions of 9 and 5 base pairs, respectively, with a dinucleotide bulge and a terminal tetraloop (most often GNRA) in the overwhelming majority of group II introns. Only a consensus sequence representing domain V may in fact be used as an initial query to scan for group II intron candidates in sequence databases [59].

**Fig. 4:**
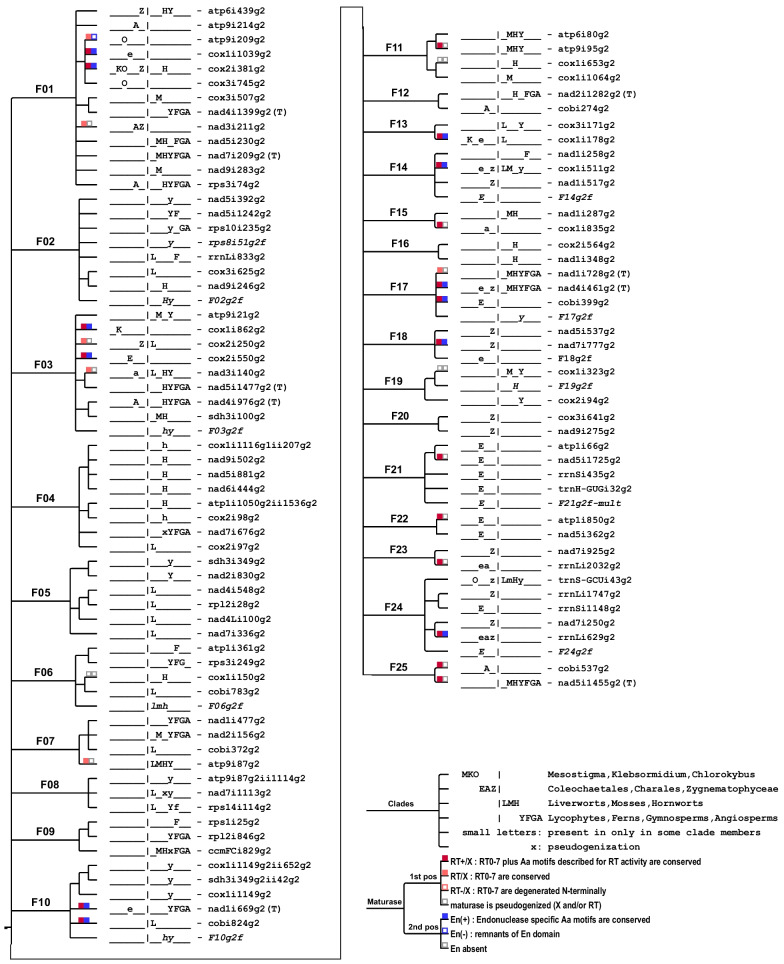
Core families F01–F25 of streptophyte mitochondrial group II introns. Cladogram of streptophyte mitochondrial group II introns categorized into core families F01-F10 (left panel) and F11-F25 (right panel) as described in the text. Occurrence of introns in the different streptophyte clades is given with a clade code before the respective intron label with underscores indicating absence in a given clade as shown in the cladogram legend (bottom right). Small letters indicate cases of possibly late intron gain within a given clade and ‘x’ indicates pseudogenization or complete loss of the respective host gene, respectively. Presence of maturases is indicated with square symbols distinguishing variable degrees of conservation of the maturase RT and X domains and the En domains as shown in the legend (bottom right). Evidently defect intron fossils are labeled with”g2f” and indicated in italics, the identification of such fossil intron inserts in intergenic sequences of mitogenomes is indicated with the respective family number

**Fig. 5:**
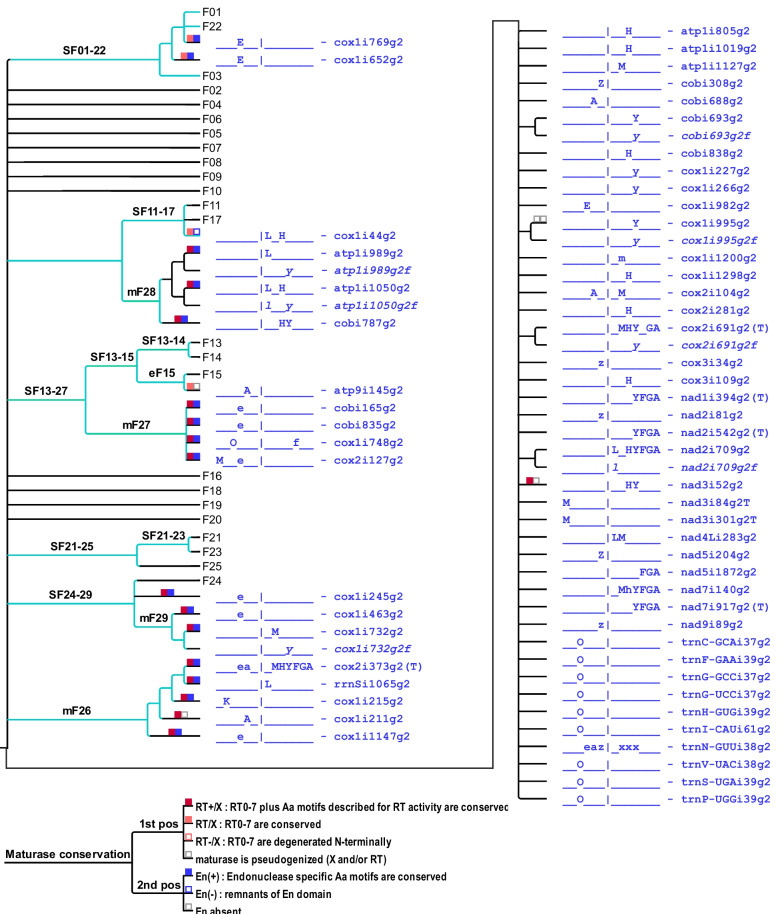
Group II intron superfamily cladogram and solitary group II introns. “Solitary” plant mitochondrial group II introns having no significant nucleotide similarities to other paralogues that could warrant inclusion in one of the core group II intron families F01–F25 (Fig. 4) are indicated in blue font. Independent phylogenetic analyses of intron-borne maturase protein sequences resulted in identification of “maturase-based” intron families mF26–m29 and superfamilies (SF) extending the core families (light blue branch lines, left panel). Symbols for maturase conservation and clade codes are as in Fig. 4

Our criteria for considering introns occupying different insertion sites for inclusion into families of related paralogues are detailed under methods. In brief, we used several rounds of identifying sequence-related intron paralogues that share significant sequence similarities (beyond short similarities of domain V and the immediate flanking regions), which can exceed those of evidently orthologous introns in the same positions in distant plant taxa. Naturally, some rare borderline cases are represented with introns occupying the same insertion site in phylogenetically very distant taxa. An independent insertion of a given position cannot be excluded as an alternative explanation to vertical transmission followed by sequence divergences obliterating recognizable similarities. Below, we will discuss such borderline cases in the context of our consideration of the now defined group II intron families. A cladogram of group II introns sorted into families is shown in Fig. 4. Subsequently, we will consider, where present, similarities of intron-borne maturases to independently verify family assignments based on the nucleotide sequence similarities alone. Furthermore, maturase similarities will occasionally allow for the inclusion of some previously “solitary” introns lacking significant nucleotide sequence similarities to other paralogues into extended families and for some fusions of primary intron families into “superfamilies” (Fig. 5).

#### Family F01

Among the here defined families of mitochondrial group II introns in the streptophyte lineage, family F01 is the largest one, comprising altogether 13 intron paralogues (Fig. 4, Additional file 3: Fig. S1). It includes four isolated introns, each presently identified in only one streptophyte algae clade (atp9i209g2 and cox3i745g2 in *Chlorokybus*, atp9i214g2 in Charophyceae and cox1i1039g2 in *Coleochaete*, respectively). The “moss-specific” introns cox3i507g2 and nad9i283g2 of F01 are universally conserved in that plant clade. Phylogenetically more broadly distributed introns atp6i439g2, nad4i1399g2, nad5i230g2, nad7i209g2 and rps3i74g2 occur in up to five embryophyte clades. In two cases, the respective introns remain recognizable despite pseudogene degeneration of *nad7* and *rps3* in hornwort mitogenomes. Three introns in family F01 are present both in at least one embryophyte clade and in up to three classes of streptophyte algae: rps3i74g2, atp6i439g2 and cox2i381g2 (Fig. 4, Additional file 3 Fig. S1). Notably though, family F01 does not include an intron paralogue present in liverworts. Only four of the intron paralogues in F01 carry maturases: atp9i209g2, cox1i1039g2, cox2i381g2 and nad3i211g2 (Fig. 4, Additional file 3: Fig. S1).

Although outgroup rooting is necessarily difficult for the individual clades of intron families, the unrooted phylogeny suggests particular close relations of certain introns in F01, in this case of the “moss” intron cox3i507g2 with the “tracheophyte” intron nad4i1399g2 and of the phylogenetically wider distributed paralogues nad5i230g2 with rps3i74g2 (Additional file 3. Fig. S1). Interestingly, the latter two and nad7i209g2 as a third intron paralogue of the F01 family have recently been demonstrated to be affected by the same nuclear-encoded splicing factor in the model angiosperm *Arabidopsis thaliana*, OZ2 [51]. Also quite notably, the maize NCS2 mutant (non-chromosomal stripe mutant) described long ago [60] is the result of a mitogenome recombination between two mitochondrial introns that we have now assigned to family F01: nad4i1399g2 and nad7i209g2.

Our later additional considerations of maturase protein similarities that we will discuss below includes family F01 into a “superfamily” also including the small family F22 and two “solitary” introns (Fig. 5).

#### Family F02

As in family F01, some intron paralogues in family F02 likewise have a very restricted occurrence: cox3i625g2 is exclusively present in liverworts and nad9i246g2 is only identified in hornworts (Fig. 4). However, despite their phylogenetically disjunct occurrence, cox3i625g2 and nad9i246g2 are closely related paralogues in F02 (Additional file 4: Fig. S2). Yet more phylogenetically restricted, nad5i392g2 is only present in the lycophyte order Lycopodiales, here represented by *Phlegmariurus squarrosus*. The extreme diversity of mitogenome evolution within the lycophyte clade with retained genes and introns and a conserved genetic synteny in the *P. squarrosus* mtDNA versus highly recombining mitogenomes with reduced gene sets in Isoetales and Selaginellales is also fully in line with intron rps10i235g2 being retained in *Phlegmariurus* and shared with the seed plants. The similarity of angiosperm rps10i235g2 to liverwort introns rrnLi833g2 and cox3i625g2 had already been noted right along with its initial discovery [61]. Other than rps10i235g2, *P. squarrosus* intron nad5i1242g2 is shared with ferns, similarly indicating an early vascular plant ancestry. Notably, we recently found intron rrnLi833g2 universally conserved among liverworts now to be also present in the mitogenome of the leptosporangiate fern *Haplopteris ensiformis* [62] and in a preliminary mtDNA assembly of the fern *Dryopteris crassirhizoma* (database accession MW732172). Intriguingly, however, the fern rrnLi833g2 introns cluster with their nad5i1242g2 paralogues, possibly indicating concerted evolution or a loss-and-regain scenario.

Intron family F02 neither includes an extant intron paralogue present in mosses nor one carrying a maturase ORF (Fig. 4). Notably though, we were able to identify several additional fossilized group II intron sequences “F02g2f” clearly tracing back to family F02 intron paralogues both in *Phlegmariurus squarrosus* and in hornwort mitogenomes (Additional file 5: Fig. S3 A & B). The series of intron copying events leading to the five F02 paralogues now recognized in the *Phlegmariurus* mitogenome (Additional file 4: Fig. S2B) remains unclear except for nad5i392g2 present only in Lycopodiales and likely emerging late from the more ancestral nad5i1242g2 paralogue. The newly identified fossil intron rps8i51g2f receives the ‘f’ behind the g2 intron label because we could not confirm functional splicing despite significant similarity to its functional counterparts in *nad5* and *rps10* extending up to the very intron 5′- and 3′-ends as opposed to another intergenic F02 intron fossil in the *tatC-cox2* spacer (Additional file 4: Fig. S2C).

#### Family F03

Intron family F03 contains two intron paralogues, nad4i976g2 and nad5i1477g2, shared between hornworts and all four tracheophyte clades, potentially supporting a hornwort-tracheophyte (HT) clade (Fig. 4). However, both nad4i976g2 and another intron paralogue in F03, nad3i140g2, also reveal counterparts in Charophyceae algae. No losses are identified for nad3i140g2 among available liverwort, hornwort or lycophyte mitogenomes. Similarly, two further intron paralogues are universally conserved in mosses and shared with either lycophytes (atp9i21g2) or hornworts (sdh3i100g2). Finally, three F03 introns have a restricted occurrence among streptophyte algae, of which only one is shared with a land plant clade, cox2i250g2 present in *Closterium* (Zygnematophyceae) and in the liverworts. This intron is an interesting exceptional case with a maturase carried in the liverwort orthologues, but not in the algal counterpart of cox2i250g2. Among the eight intron paralogues in F03 (Fig. 4), two pairs of introns show particularly high sequence similarities: nad3i140g2 with nad5i1477g2 and sdh3i100g2 with nad4i976g2. In this case, the intron paralogues occur with a phylogenetic overlap in the hornworts, a clade characterized by high intron mobility as also reflected below with the example of family F04. Again, we were able to identify fossilized group II intron sequences (“F03g2f”) in intergenic sequences of the lycophyte *Phlegmariurus squarrosus* clearly tracing back to family F03 (Additional file 5: Fig. S3C).

#### Family F04

Group II intron family F04 is dominated by introns exclusively occurring in hornworts, indicating a pronounced intron mobility in that land plant clade: cox2i98g2, nad5i881g2, nad6i444g2 and nad9i502g2 (Fig. 4). This is further enhanced by the observation that the respective internal introns of two recently discovered twintrons [14], atp1i1050g2ii1536g2 and cox1i1116g1ii207g2 in the hornwort genus *Anthoceros,* likewise belong to the here defined F04 family. In fact, the extraordinary sequence similarity of 98% between atp1i1050g2ii1536g2 to cox2i98g2 is the most striking case of closely related paralogues in our entire intron sampling, indicating a very recent copying event. Considering that the *atp1* twintron is present in all hornworts whereas cox2i98g2 exists only in the genus *Anthoceros* immediately suggests a copying from the former to the latter insertion site. Notably, however, internal splicing of zombie-twintron atp1i1050g2ii1536g2 could not be detected.

At the same time, family F04 is a prime example for the necessity of careful analyses to distinguish cox2i98g2 from its paralogue cox2i97g2 in liverworts that is inserted one nucleotide upstream in the *cox2* gene (Fig. 2). Intron nad7i676g2 is conserved in all tracheophyte clades. Its former presence in hornworts, and accordingly in a possible HT stem lineage, is elusive owing to the pseudogene degeneration of the downstream part of *nad7* in all hornwort mtDNAs. Notably, and despite the very clear evidence of recently active retrotransposition, none of the F04 members carries a maturase-ORF (Fig. 4).

#### Family F05

In contrast to F04 mainly comprising intron paralogues in hornworts, group II intron family F05 (Fig. 4) is dominated by intron paralogues exclusively occurring in liverworts: nad4i548g2, nad4Li100g2, nad7i336g2 and rpl2i28g2. Liverwort-specific intron nad4i548g2 had been introduced to elucidate the liverwort phylogeny [63] and is now found to be notably similar to rpl2i28g2. Other than the four liverwort paralogues, F05 also includes lycophyte introns recently discovered and characterized as the external intron of a “zombie” twintron upon closer reinspection of the *Phlegmariurus squarrosus sdh3* gene [14].

#### Family F06

Apart from rps3i249g2 that is shared between lycophytes, ferns and gymnosperms, the group II intron paralogues in family F06 are phylogenetically very restricted in occurrence: atp1i361g2 exclusively in ferns, cobi783g2 only in liverworts and cox1i150g2 exclusively in hornworts (Fig. 4). A detailed study of atp1i361g2 concluded that this fern-specific intron has originated from the, likely more ancestral, paralogue rps3i249g2 and also found evidence for convergent evolution of specific intron structures, mainly group II intron domain III, in the two paralogues in later emerging fern lineages [26].

Interestingly, a truncated copy of cobi783g2 had been identified earlier in the spacer between *nad5* and *nad4* in liverworts in an early sampling of this intergenic region among bryophytes [64]. Our extended consideration of intron families now adds support to the idea that also this case of an intron fossil has arisen through copying, likely by a retrotransposition event. Similarity of the fossil sequence starts exactly from the intron 5’-end and extends for nearly 800 bp with 99% identity in the case of *Treubia lacunosa* representing an early liverwort branch (Additional file 5: Fig. S3D) whereas a higher degree of degeneration is observed in derived taxa. Yet other intron fossils have now been discovered in the intergenic region between *rrn5* and *trnM*-CAU of the hornwort *Anthoceros agrestis* and the moss *Sphagnum palustre* mitogenomes (Additional file 5: Fig. S3 E). The only evidence for a maturase among the four members of intron family F06 are traces of a former maturase-ORF in cox1i150g2.

#### Family F07

Intron family F07 contains three paralogues of phylogenetically wide distribution and only one member with isolated occurrence in the liverworts alone: cobi372g2 (Fig. 4). The similarity between introns nad1i477g2 and nad2i156g2, now found to be widely conserved among vascular plants (Fig. 4), has been recognized early after the complex structures of *nad1* and *nad2* in flowering plants had been elucidated [55]. Quite interestingly, the nuclear encoded splicing factor ODB1 has meantime been found to promote splicing of both of these two paralogues [65]. The fourth paralogue in family F07, intron atp9i87g2, is particularly noteworthy for (i) being an intron now identified to be shared between all three bryophyte clades, (ii) carrying an ancestral maturase that has independently degenerated in all four plant clades where it is present and (iii) carrying the internal intron atp9i87g2ii1114g2 of family F08 (see below) turning it into a twintron in the lycophyte *Phlegmariurus squarrosus*. A history of intron paralogues is immediately suggestive for the family F07 paralogues (Additional file 6: Fig. S4). The ancestral maturase-carrying atp9i87g2 independently likely gave rise to cobi372g2 in liverworts and to nad2i156g2 in a possible non-liverwort lineage. After loss of nad2i156g2 in hornworts, it gave rise to nad1i477g2 only in the tracheophyte lineage.

#### Family F08

Two introns in family F08 have a phylogenetically striking distribution being conserved in liverworts but also occurring in lycophytes: nad7i1113g2 and rps14i114g2 (Fig. 4). Given that the host genes, *nad7* and *rps14*, are frequently subject to EGT, nad7i1113g2 is at present only determined in *Isoetes engelmannii* and rps14i114g2 only in *Phlegmariurus squarrosus* among the lycophytes. However, we now found rps14i114g2 equally conserved in the mitogenome of the leptosporangiate fern *Haplopteris ensiformis* [62]. Yet more notably, the recently characterized inner intron of a twintron in the *atp9* gene, atp9i87g2ii1114g2 [14] as a third member in family F08 is characteristically more similar to its rps14i114g2 counterpart in liverworts than in *P. squarrosus*.

#### Family F09

Intron ccmFCi829g2 in F09 has a phylogenetically wide distribution in bryophytes and tracheophytes (Fig. 4). Its former presence in lycophytes remains unclear, however, owing to the loss of the entire *ccm* gene suite for cytochrome c maturation in this clade. In contrast, pseudogene traces of *ccmFC* including ccmFCi829g2 are clearly detected in hornworts [66]. Intron rpl2i846g2 is evidently a gain in the tracheophyte stem lineage. This ancestral intron obviously gave rise to its closely related paralogue rps1i25g2 exclusively present in ferns [25]. As in family F08, no traces of maturase-ORFs are recognizable in any of the F09 intron paralogues. Highly interesting, however, the splicing of both F09 paralogues present in *Arabidopsis thaliana*, ccmFCi829g2 and rpl2i846g2, was found to be affected by the same nuclear-encoded splicing factor, WTF9 [67].

#### Family F10

Intron family F10 comprises group II introns with a particularly complex history (Fig. 4). It contains two intron paralogues with a phylogenetically disjunct distribution: cobi824g2 exclusively present in liverworts and nad1i669g2 previously assumed to be restricted to tracheophytes. Intron nad1i669g2 has received attention as being conserved in a *trans*-splicing arrangement in seed plants. A *cis*-arranged orthologue was initially identified in the fern *Osmunda regalis*, also noticing traces of a degenerated maturase [6]. We now found that intron nad1i669g2 has a clear orthologue also in the mtDNA of the alga *Coleochaete scutata* [68] with a maturase-ORF annotated in the corresponding database entry (MN613583). Intron family F10 also contains the “hypermobile invader” intron cox1i1149g2 in the lycophyte *Phlegmariurus squarrosus*, which gave rise to internal introns of two twintrons (in the newly determined sdh3i349g2 and in itself) and to seven further intron fossils in intergenic regions [14] (Additional file 5: Fig. S3 F).

#### Family F11

Intron family F11 contains introns atp6i80g2 and atp9i95g2 of phylogenetically wider distribution, shared between mosses, hornworts and lycophytes (Fig. 4). Like the above cases of the neighboring introns cox2i97g2 and cox2i98g2 in F04, also atp9i95g2 requires careful inspection given the closely neighboring intron atp9i87g2 in family F07 [66]. Despite the evident dynamics of intron insertions in *atp9* of bryophytes and lycophytes, no evidence is ever found for an intron in the *atp9* gene among ferns, gymnosperms or angiosperms. Intron cox1i1064g2 is exclusively present in mosses and cox1i653g2 is only present in hornworts. Again, the latter needs particular attention to distinguish it from an unrelated “solitary” intron cox1i652g2 inserted one nucleotide upstream in the *Coleochaete* mitogenome.

#### Family F12

The two intron paralogues in family F12 (Fig. 4) have a strikingly divergent distribution with cobi274g2 presently only identified in Charales algae and nad2i1282g2 present in hornworts and euphyllophytes (i.e., ferns and seed plants). No traces of former maturase reading frames can be detected in nad2i1282g2 or cobi274g2.

#### Family F13

Group II intron family F13 contains two intron paralogues conserved in liverworts: cox1i178g2 and cox3i171g2 (Fig. 4). The former has counterparts in the algae *Klebsormidium flaccidum* and *Coleochaete scutata*. However, only cox1i178g2 in *Coleochaete* shares significant sequence similarity with the liverwort homologues, clearly warranting family inclusion according to our criteria. The “positional homologue” in *Klebsormidium* neither shares similarity with the liverwort nor with the *Coleochaete* counterpart, leaving its status as a true orthologue vs. a possible “analogue” occupying the same insertion site open at present. The cox1i178g2 introns carry maturase reading frames both in algae and the liverworts, no intron-borne ORFs are present in the much smaller cox3i171g2 paralogues shared between liverworts and the lycophyte *Phlegmariurus squarrosus*.

#### Family F14

As in family F12, the intron members in family F14 are also very disjunct in occurrence with nad1i258g2 being restricted to the monilophyte (fern) clade and nad1i517g2 so far only identified in the alga *Zygnema circumcarinatum* (Fig. 4). The third paralogue, cox1i511g2, however, is present in liverworts, mosses, lycopyhtes and the algae *Coleochaete* and *Zygnema*. Like most group II introns in the algal mitogenomes also cox1i511g2 carries maturase reading frames. The counterparts in the land plant lineage, however, are frameshifted ORFs in liverworts and degenerated or unrecognizable in the cox1i511g2 orthologues of mosses or in the lycophyte *Selaginella moellendorffii*. We identified an F14-type intron fossil (F14g2f) in the *cox1-rrnS* spacer of the *Coleochaete* mitogenome (Additional file 5: Fig. S3 G).

#### Family F15

As in F14, the intron paralogues in family F15 are likewise phylogenetically disjunct (Fig. 4). Intron cox1i835g2 is presently only identified in the algal genus *Chara* (and absent in the Characeae genera *Nitella* and *Nitellopsis*). Intron nad1i287g2 had initially been identified serendipitously in a screen for ancestors of *trans*-splicing intron nad1i394g2 [6] and is meantime found to be universally conserved both in mosses and in hornworts [57, 66]. As in the above cases, a maturase reading frame is only present in the algal cox1i835g2 but not in the much smaller nad1i287g2 paralogues in the bryophytes of less than 800 bp. The subsequent additional analyses of intron-borne maturases (see below) add S-type intron atp9i145g2 in Charales to an extended family eF15, which is ultimately linked to the superfamily SF13-14 (Fig. 5).

#### Family F16

Family F16 contains two intron paralogues that occur exclusively in hornworts: cox2i564g2 and nad1i348g2 (Fig. 4). The latter intron is of exceptionally small size of less than 600 nt. and conserved in all hornwort genera. In contrast, cox2i564g2 is ca. five times larger and lost together with the upstream and downstream neighboring introns cox2i381g2 and cox2i691g2 in *Nothoceros aenigmaticus*. Despite their extended sizes of more than 2.5 kb no maturase traces are discernible in the hornwort cox2i564g2 copies.

#### Family F17

Intron family F17 contains two intron paralogues with a phylogenetic distribution that could have been taken as further support for an NLE (“Non-Liverwort Embryophyte”) clade: nad1i728g2 and nad4i461g2 (Fig. 4). Both introns are absent in liverworts but particularly well conserved in mosses, hornworts, lycophytes and vascular plants with only rare exceptions including the absence of nad4i461g2 in the hornwort *Leiosporoceros dussii* and of nad1i728g2 in the lycophyte *Isoetes engelmannii*. Intron nad4i461g2 counterparts in the algae *Coleochaete scutata* and *Zygnema circumcarinatum* are large introns of 3.1 and 5.7 kb with long maturase reading frames that are continuous with the upstream *nad4* coding sequence. Matching the general observations, only small traces of former maturases remain in the land plant counterparts where the sizes of nad4i461g copies in the mosses are reduced to less than 800 bp.

Intron nad1i728g2 is a particularly interesting case, being the only mitochondrial intron carrying a maturase reading frame in flowering plants, widely known as “matR”, now systematically labelled *mat-nad1i728g2*. Moreover, intron nad1i728g2 is also known for multiple independent transitions from *cis*- to *trans*-splicing with intron disruptions either up- or downstream of the maturase ORF in flowering plants [7]. Intriguingly, a gene transfer of the *matR/mat-nad1i728g2* reading frame into the nuclear genome has been identified in *Pelargonium* [69]. Most interestingly, three different nuclear-encoded splicing factors have already been identified, which affect the two closely related F17 angiosperm paralogues nad1i728g2 and nad4i461g2 simultaneously: EMP8 [70], DEK55 [71] and SMK3 [72]. The third paralogue in F17, cobi399g2, is so far only identified in the *Coleochaete scutata* mitogenome and, as in most cases in the algal mitogenomes, also carries a maturase reading frame. The intergenic region between *trnM-CAU* and *trnA-UGC* contains an F17-type intron fossil (F17g2f) in the *Phlegmariurus squarrosus* mitogenome (Additional file 5: Fig. S3 H)*.*

#### Family F19

Group II intron family F19 (Fig. 4) comprises two introns present in lycophytes where sequence similarities are blurred by the highly divergent mitogenomes in genera *Isoetes* and *Selaginella* and largely rely on the conserved mitogenome of *Phlegmariurus squarrosus*. While intron cox2i94g2 is exclusively present in all three orders of lycophytes, intron paralogue cox1i323g2 is also present in the mitogenome of *Sphagnum*, representing a very early branch in the phylogeny of mosses. In both, *Phlegmariurus* and *Sphagnum* cox1i323g2 contains maturase remnants. Notably, cox1i323g2 has extensive similarity with the extended intergenic region between *nad9* and *trnI-CAU* in hornwort mtDNAs, representing yet another example for traces of an intron fossil (Additional file 5: Fig. S3 I).

#### Family F24

Intron family F24 comprises rrnSi1148g2 present in *Coleochaete scutata*, rrnLi1747g2 presently only identified in *Entransia fimbriata,* nad7i250g2 present in the Zygnematophyceae algae *Closterium baillyanum* and *Gonatozygon brebissonii*, rrnLi629g2 present in *Entransia*, *Coleochaete* and *Nitella* and trnS-GCUi43g2, the only F24 paralogue shared with embryophytes (Fig. 4). The latter is present in *Chlorokybus* and the Zygnematophyceae genera *Closterium, Gonatozygon* and *Roya* and highly conserved among liverworts. Moreover, trnS-GCUi43g2 is evidently present as a degenerated copy in the *trnS-GCU* pseudogene retained in the conserved intergenic space between *trnA* and *trnD* in the mitogenomes of mosses and the lycophyte *Phlegmariurus squarrosus*. An independent degeneration has occurred among hornworts with functional copies present in *Leiosporoceros* and *Anthoceros* but pseudogenes in *Nothoceros* and *Phaeoceros*. Hence three independent degenerations of *trnS-GCU* and its intron are evident in land plants in mosses, among hornworts and in the tracheophytes (Additional file 5: Fig. S3 K).

#### Family F25

Family F25 comprises two intron paralogues of phylogenetically disjunct distribution: cobi537g2 present in the Charales algae and nad5i1455g2, present in all embryophytes except the liverworts (Fig. 4).

#### Group II intron families presently restricted to streptophyte algae

Five group II intron families are currently represented only by paralogues restricted in occurrence to the streptophyte algae: F18, F20, F21, F22 and F23 (Fig. 4). Given that owing to the presently much wider sampling of land plant mitogenomes the current focus of our work is on land plants, we discuss those five families separately in text Additional file 1.

### Maturases in the plant mitochondrial lineage and extended maturase-based intron families

Our categorization of 100 group II introns into the 25 “core” families F01–F25 outlined above is based on their primary nucleotide sequence similarities alone. To cross-check for independent confirmation and to explore further and deeper relationships, also outside of the streptophytes, we independently compiled the intron-borne maturase ORFs present in 43 streptophyte mitochondrial group II introns as seeds for identifying protein homologs. This seed query data set also included several maturases that remained hitherto unnoticed or not annotated in database entries (e.g., the spliced variants of *mat-atp9i87g2* and *mat-atp9i95g2* in *Phlegmariurus*). The search for homologs ultimately resulted in a large protein data set that also contained related maturases of distant chlorophyte algae as well several maturase proteins in red algae, stramenopiles, fungi, animals and bacteria. The independent phylogenetic analysis of the large maturase protein sequence collection (Additional file 7: Fig. S5) fully confirmed the identified core families F01, F03, F10, F11, F17 and F25, all of which contain at least two intron paralogues carrying maturases (Fig. 5). Moreover, the maturase similarities identified four additional, “maturase-based” group II intron families mF26-mF29 and helped to define superfamilies (SF) of higher order that combine the core intron families and include additional, previously solitary, introns.

**Fig. 6:**
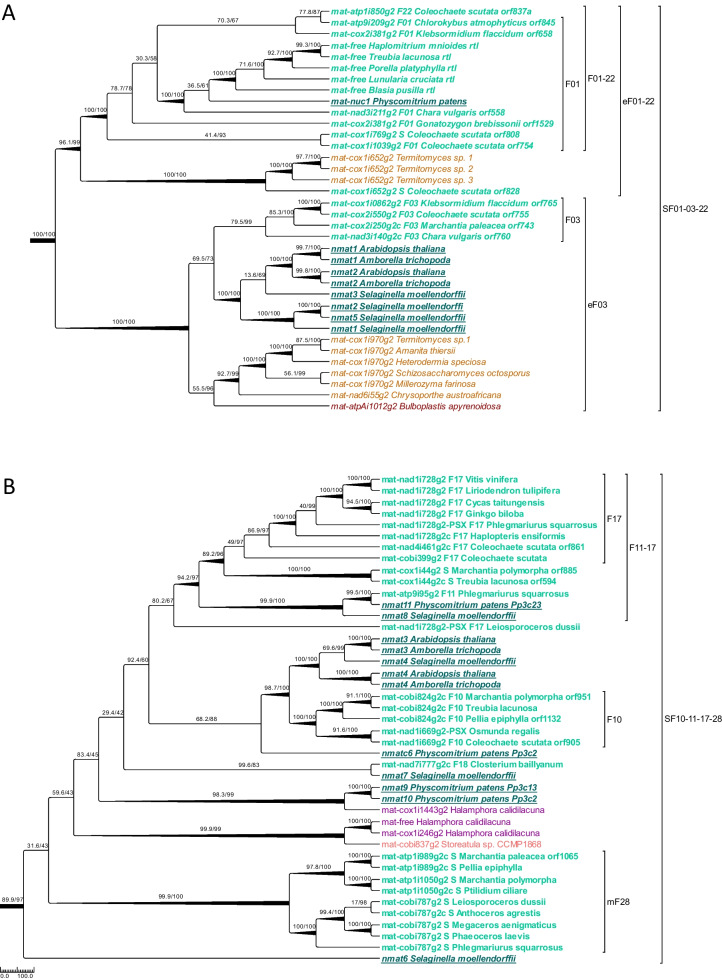
Group II intron superfamilies defined by maturase similarities. Two selected subclades of the comprehensive maturase phylogeny shown in Additional file 7: Figure S5 for group II intron superfamilies SF01-03-22 (**A**) and SF10-11-17-28 (**B**). The eukaryote maturase phylogeny contains samples from nuclear encoded maturases of embryophytes (dark green with underline), streptophyte mitochondria (green and bold), fungal mitochondria (orange), metazoan mitochondria (blue) and organelles of chlorophytes (light green), stramenopiles (purple), cryptophytes (beige), rhodophytes (red) and oomycetes (black). **A** SF01-03-22 contains solitary-type streptophyte mitochondrial maturase *mat-cox1i769g2* and *mat-cox1i652g2*, which forms a joint clade with orthologs from fungal *Termitomyces* mitochondria*.* The free-standing mitochondrial maturases of liverworts (“mat-free”) cluster with the nuclear encoded mat-nuc1 of *P. patens* and with maturases of algal F01 paralogs while no F01 intron paralogues are present in liverwort mitogenomes. Extended family eF03 supports a close relation of F03 paralogs and the well-characterized nuclear-encoded maturases nmat1 and nmat2 of angiosperms together with nuclear maturase paralogs in the lycophyte *S. moellendorffii* and, more distantly, with mitochondrial maturase paralogs in fungi and a chloroplast paralogue in a rhodophyte.** B** Superfamily SF10-11-17-28 contains F17 *mat-nad1i728g2 (*or *matR),* the only mitochondrial encoded maturase in euphyllophytes together with the maturases in the S-type intron cox1i44g2 and *mat-atp9i95g2*. Angiosperm nuclear encoded nmat3 and nmat4 form a joint clade with maturases of F10 paralogs including the pseudogenized (PSX) *mat-nad1i669g2* in the fern *Osmunda regalis* indicating the ancestral presence of a maturase in this intron paralogue conserved also in angiosperms. Maturases of S-type introns *mat-atp1i1050g2* and *mat-atp1i989g2* form a well-supported “maturase family” clade mF28 with *mat-cobi787g2* as sister group

For space limitations, we here focus on the examples of the large superfamily SF01-22 comprising families F01, F03, F22 and the two previously solitary introns cox1i652g2 and cox1i769g2 (Fig. 6A) and on superfamily SF10-28 comprising families F10, F17 and mF28 (Fig. 6B). The independent protein analysis fully confirms monophyly of the maturases in F03 and a well-supported clade of maturases in F01, now extended to include to mat-cox1i769g2, mat-atp1i850g2, the free-standing maturases in the mitogenomes of liverworts and a nuclear maturase copy (mat-nuc1) in the moss *Physcomitrium* (Fig. 6A). Notably, the extended SF01-22 superfamily also includes homologs in fungi having identical insertion sites and clustering with *mat-cox1i652g2* in *Coleochaete* with high support. Vice versa, the extended F03 maturase clade likewise includes fungal mitochondrial maturases and a rhodophyte plastid maturase and, maybe even more notable, a cluster of nuclear maturases in tracheophytes (Fig. 6A).

As in the above case, the independent maturase phylogeny perfectly confirms the intron assignments to families F10 and F17 and adds the maturase-based family mF28 for a joint inclusion in superfamily SF10-28 (Fig. 6B). Particularly intriguing further cases for introns giving rise to fossil paralogues are “liverwort” introns atp1i989g2 and atp1i1050g2 (also present in hornworts), which are now jointly placed in mF28 (Figs. 4, 6B). An extended and significant sequence similarity (with perfect intron domain V and VI ends) of atp1i989g2 is present in the mitogenome of the lycophyte *Phlegmariurus squarrosus* embedding the *trnI-rps11* region with all nine genes in the same direction fitting the intron orientation (Additional file 5: Fig. S3 L). Hence, it appears that a huge block of genes was inserted into an intergenic intron fossil paralogue of atp1i989g2. Interestingly, intron atp1i989g2 is absent in the early branching liverwort genera *Treubia* and *Haplomitrium*, which could have indicated a gain within the liverworts only after split of the Haplomitriopsida, but this now seems unlikely given the unorthodox *Phlegmariurus* fossil paralogue. Along the same lines, intron atp1i1050g2 has fossil intron paralogues not only in liverwort mitogenomes but also in the *Phlegmariurus* mtDNA behind *cox1* in the spacer towards the *trnW* gene running in the opposite direction (Additional file 5: Fig. S3M).

A further, very notable insight emerges from the maturase phylogeny: Mitochondrial group II introns with intron-borne maturases at the same insertion sites appear distributed across very distantly related lineages of eukaryotes. The most striking example concerns group II introns inserted into position 1147 of the *cox1* gene. Solitary type intron cox1i1147g2 inhabits mitogenomes of the streptophyte alga *Coleochaete,* but also in chlorophytes, rhodophytes, fungi and metazoa. The associated *mat-cox1i1147g2* RT-domains, the X-domain and the D/En domain are highly conserved. Similarly, the peculiar case of cox1i748g2 in *Equisetum arvense* [73], but not *E. diffusum*, having no similarity to its *Chlorokybus* counterpart is also found in the brown alga *Pylaiella littoralis* and the red alga *Pyropia fucicola.* A third intriguing example along those lines is cox2i373g2 of *Coleochaete* (now placed in mF26) that has maturase-free orthologs in mosses, hornworts and tracheophytes but forms a well-supported maturase-based clade with *mat-cox2i373g2* in ascomycetous fungi, e.g., the endophytic symbiont *Epichloe.*

### The remaining “solitary” introns lacking significantly similar paralogs

Altogether 61 streptophyte group II introns lacked paralogues with significant nucleotide sequence similarity precluding their assignment into our 25 core families. Extending the analysis to characteristic maturase similarities placed 15 of them in the four additional families mF26-mF29 and included another four previously solitary introns into superfamilies (cox1i44g2, cox1i245g2, cox1i769g2 and cox1i652g2). This leaves 42 streptophyte mitochondrial group II intron solitary, lacking both primary nucleotide similarity to paralogues and an intron-borne maturase of significant similarity to protein homologues (Fig. 5 and Additional file 2).

## Discussion

### Group II intron mobility outside of land plants

It is reasonable to assume that an ancestral state of group II introns with fully equipped intron-borne maturases is at the origin of their mobility and diversity (Fig. 1A). After the first discoveries of group II introns in bacteria [59, 74–76], their collection has grown immensely [77]. Bacterial group II introns are frequently associated with mobile genetic elements and/or present on plasmids [78] whereas their presence in essential genes is quite rare in bacteria [79]. Accordingly, their routes of dispersal are somewhat hard to trace and additionally complicated by the generally abundant horizontal gene transfer (HGT) among prokaryotes. Notably, prokaryotic group II introns lacking maturases are rare with “only a handful of ORF-less introns in bacteria” [80] and the few examples suggest a (likely quick) degeneration from maturase-bearing counterparts (Fig. 1B).

It should be noted that a “family” terminology for introns has already been used earlier in scoring bacterial group II intron occurrences and their retroelement behavior [80–82]. Among the research on group II introns in the bacterial world [83], the studies of mobile group II introns in *Wolbachia* endosymbionts [84] or of the highly mobile RmInt1 intron shaping the genome of *Sinorhizobium meliloti* and related α-proteobacteria [85] are typical cases in point on low taxonomic levels.

Several reports on variability, and likely mobility, of group II introns have also been published for eukaryotic organelle genomes, e.g. for the chloroplast genome of *Euglena* species [86], for mitochondria of different isolates of the brown alga *Pylaiella littoralis* [87] or in chloroplasts and mitochondria of the red algal genus *Porphyridium* [88, 89]. On somewhat higher taxonomic levels, significant group II intron diversities have been reported for the plastid genomes of cryptophytes [90] or the mitogenomes of diatoms [91, 92]. Extraordinary similarity of cyanobacterial introns in *Porphyra* [93] or the chloroplast of *Chlamydomonas* [94] or *Euglena* [95] have been discussed as horizontal transfer events. Likewise, horizontal transfers were also invoked as the likely cause in other cases of striking intron similarities, e.g. between those of diatoms and a haptophyte [96], for diatoms as likely group II intron donors into a raphidophycean flagellate genus *Chattonella* [97] or to explain the discovery of a first group II intron in a bilaterian mitogenome [98].

### The plant mitochondrial group II intron family concept

The extraordinary dynamics of plant mitochondrial introns is in stark contrast not only to the overall stability of the chloroplast intron complements [99, 100], but also to the stasis of nuclear introns since 500 million years and more [101]. Many review articles [e.g. 22, 102] and reports on newly completed plant mitogenomes, respectively, often contain comparative summaries and updates on the striking diversity of plant mitogenome makeups with respect to their gene and intron complements [12, 13, 66, 103].

Here, we have added on the previous comparative compilations by providing the hitherto most extensive overview on mitochondrial group II introns by including mitogenome analyses of all embryophyte and streptophyte algae lineages, also taking care of pseudogenization events and the existence of fossil introns in intergenic spacer regions. More importantly, we provide a concept for categorizing group II introns into families of related paralogs as a foundation to explore intron “copying” retrotransposition events during plant evolution. Of altogether 161 streptophyte mitochondrial group II introns, we have assigned 100 into 25 “core” families of minimally two and up to 13 intron paralogues based on their nucleotide sequences alone (Fig. 4). The family assignments may certainly be subject to further changes with newly discovered mitochondrial group II introns in streptophytes added to the 25 defined families or with new families or superfamilies (Fig. 5) being created by newly identified introns linking existing families. While we do not expect many more introns to be discovered in the land plant (embryophyte) lineage, the highly diverse streptophyte algae are presently still underrepresented in the sampling for mitogenomes with only twelve genera representing the five classically distinguished classes and will likely reveal many more intron paralogues in the future.

**Fig. 7:**
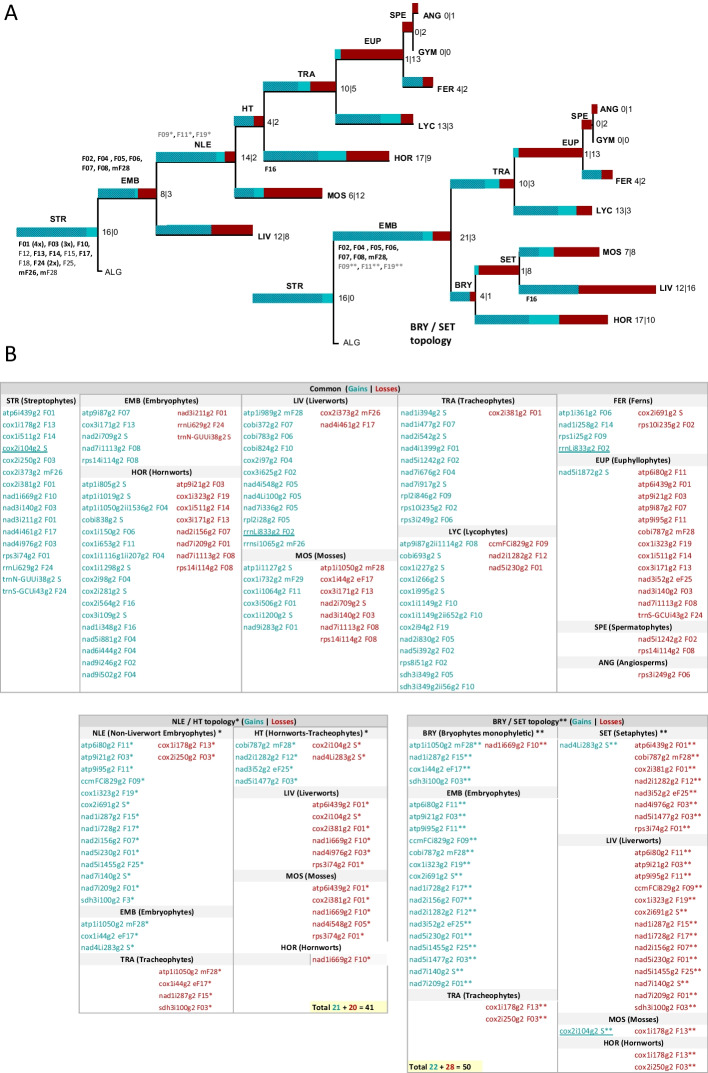
Possible gain–loss-scenarios for plant mitochondrial group II introns. **A** Cladograms for alternative phylogenies “NLE/HT” (Non-Liverwort Embryophyte and Hornwort-Tracheophyte clades, left) and alternative “BRY/SET” (monophyletic Bryophyte and Setaphyte clades, right). Further labels indicate Streptophytes (STR), Embryophytes (EMB), Liverworts (LIV), Mosses (MOS), Hornworts (HOR), Lycophytes (LYC), Tracheophytes (TRA), Euphyllophytes (EUP), Ferns (FER), Gymnosperms (GYM), Angiosperms (ANG), Spermatophytes (SPE), and Streptophyte algae (ALG). Bars indicate plant mitochondrial group II intron gains (blue) and losses (red) after search for maximum parsimony evolution for the distribution of 101 embryophyte mitochondrial group II introns assuming weights of 4 to 1 for gains over losses. Underlining of rrnLi833g2 and cox2i104g2 highlights likely independent gain events as discussed in the text. Numbers before and after the pipes indicate intron gain and loss events. Hatched blue areas indicate introns grouped into families and the origin of the respective families are indicated on the respective branches. **B** Detailed listing for gains and losses of the embryophyte group II introns with those common to both evolutionary scenarios on top and those that need to be specifically assumed for the NLE/HT scenario (asterisks, total 102 gain and 190 loss events) shown below to the left and those for the BRY/SET scenario (double asterisks, total 103 gain and 199 loss events) shown below to the right

Somewhat surprisingly, our intron inventory does not reveal a particular strong affiliation of land plants with Zygnematophyceae algae, currently considered to be the sister lineage of embryophytes despite this class being best sampled for mitogenomes with five genera representing four families. Similarly, mitochondrial group II introns have contributed to a model phylogeny of land plants assuming liverworts as sister to all other embryophytes and hornworts as sister to tracheophytes [9, 104, 105]. Alternative datasets, however, now favor the concept of monophyletic bryophytes with a sister group relationship of mosses and liverworts [106–109]. Both phylogenies must postulate massive gains and losses of mitochondrial group II introns in the early embryophyte lineages (Fig. 7). Testing the alternative phylogenetic concepts by Maximum Parsimony searches for the 101 mitochondrial group II introns present in embryophytes, we still find weak support for the NLE/HT topology requiring nine steps less than the monophyletic Bryophyte topology (Fig. 7).

Our dedicated scan for group II intron fossils indicates that retrotransposition has evidently contributed to the expansion of intergenic spacers in the mitogenomes of some taxa quite significantly. Their detailed investigation in the characteristically inflated mitogenomes of the alga *Coleochaete scutata*, the hornwort *Anthoceros agrestis* and the lycophyte *Phlegmariurus squarrosus* reveals yet more fragmented intron fossils that fell beyond our initial inclusion into intron families. Altogether, the recognizable intron fossils contribute to 3–4% of mtDNA size in these mtDNAs (Additional file 8: Table S1).

### Similarities of intron paralogues in the absence of intron-borne maturases

The dynamics of group II introns in plant mitochondrial vs. chloroplast genomes is puzzling at first sight. Key to an explanation for this observation may be the co-evolution of group II introns with concomitant splicing factors and the ancestrally intron-borne maturases certainly play an initial key role. In flowering plants, only one maturase each remains present in the organelle DNAs—“MatK” in the group II intron disrupting the *trnK* gene (mat-trnKi37g2) and “MatR” in the terminal group II intron of the *nad1* gene of mitochondria (mat-nad1i728g2). Accordingly, the lack of evidently recent group II intron mobility in angiosperm organelles, and in fact even spermatophytes, is likely no coincidence. Notably, whereas MatK is also the only chloroplast maturase conserved in the other land plant clades, many more mitochondrial maturases exist in early land plant lineages (Figs. 4 and 5), which have likely contributed to intron retrotranspositions during establishment of the ancestral plant lineages. Additionally, four nuclear-encoded maturases (Fig. 1C) have been shown to functionally affect diverse mitochondrial, but not chloroplast, group II introns in *Arabidopsis* [16–21].

In some cases, it appears indeed suggestive that a maturase-containing intron paralogue is the likely source of the other paralogues in a family. Key examples are the large intron families F01 and F03 where five out of 13 and four out of eight paralogues, respectively, carry maturases at least in taxa representing early branching lineages (Fig. 4). Other examples are family F07 with its likely ancestral “mother intron” atp9i87g2, family F10 with cobi824g2 or nad1i669g2, family F13 with cox1i178g2 and family F14 with cox1i511g2 carrying a maturase in the alga *Coleochaete scutata*.

Vice versa, however, no traces of maturases are discernible in many other families (Fig. 1F) and even despite the existence of related paralogues in the same plant lineages such as the “liverwort-lycophyte” family F05, the “liverwort-lycophyte” family F08, the “moss-hornwort” family F11, the “hornwort” family F16 or the large and diverse family F02 (Fig. 4). Other striking examples include family F06 where it is highly likely that atp1i361g2 has arisen from rps3i249g2 exclusively in ferns, family F09 where the same holds true for rps1i25g2 likely originating from rpl2i846g2 and family F04 dominated by hornwort paralogues including recently emerged twintron arrangements (Fig. 4). The latter represents the most intriguing case of 98% sequence similarity between the maturase-free paralogues atp1i1050g2ii1536g2 and cox2i98g2, indicating a very recent origin of cox2i98g2 in the genus *Anthoceros* originating from the internal intron of the *atp1* twintron present in all hornwort mitogenomes.

Similarly, the lack of intron-borne maturases holds true for most of the solitary, S-type introns lacking any evident similarity to other introns (Fig. 5). Only 14 of 61 S-type introns show up in more than one plant clade and any evidence for an external origin via HGT is missing. Like the above cases of maturase-less, but clearly related, paralogues their origin from maturase-bearing ancestors would necessitate a surprisingly quick degeneration of intron-borne reading frames that is incompatible with the overall slow sequence evolution in plant mitogenomes, which reveal the retention of pseudogene traces including former maturases in many other cases [15, 110, 111]. The numerous examples of group II intron fossils reported here are a further case in point (Fig. 1G). After an early discovery of a degenerate group II intron in *Chlamydomonas* [112] this issue may have received less attention than deserved in the exploration of newly determined organelle genomes.

The participation of external protein factors rather than only intron-borne maturases promoting retrotransposition, possibly including nuclear-encoded maturases in the early plant lineages [113], seems a more likely explanation for early group II intron propagations in plant mitochondria (Fig. 1E).

### Nuclear splicing factors acting on paralogues in the same family

Quite interestingly, we found that in several cases nuclear-encoded splicing factors have already been identified (Fig. 1E), which simultaneously act on related intron paralogues that we have now grouped into families. The nuclear-encoded splicing factor ODB1 (“Organelle DNA Binding 1”) containing a RAD52-like domain has been found to promote splicing of the two angiosperm intron paralogues in family F07, nad1i477g2 and nad2i156g2 [65]. Likewise, the two F09 introns present in angiosperms, ccmFCi829g2 and rpl2i846g2, depend on WTF9, a splicing factor containing a PORR (Plant Organelle RNA Recognition) domain [67]. In family F01, even three different intron paralogues present in angiosperms (nad5i230g2, nad7i209g2 and rps3i74g2) rely on splicing factor OZ2, a RanBP2-type zinc finger protein [51]. Finally, the two F17 introns present in angiosperms (nad1i728g2 and nad4i461g2 have also been shown to both depend on three different splicing factors: the two pentatricopeptide repeat proteins DEK55 [71] and EMP8 [70] and the mTERF-type (Mitochondrial Transcription Termination Factor) protein SMK3 [72]. It will be highly interesting to see whether the splicing factor functionalities demonstrated in the model angiosperm species *Arabidopsis thaliana* or *Zea mays* are conserved deep in flowering plant evolution or even beyond angiosperms once adequate model taxa are established among gymnosperms, ferns and lycophytes.

## Conclusions and perspectives

Possibly even more interesting for the future developments will be whether nuclear factors like the ones discussed above and others yet to be identified, aside from their evident function in facilitating proper RNA structures for forward-splicing, may also have contributed to the ancient retrotranspositions creating the related paralogues. Functional studies on plant mitochondrial group II introns will necessarily rely on heterologous experimental systems to detect splicing and retromobility, ideally in easily amenable genetic systems like *Escherichia coli* that has recently been successfully established for investigation of plant RNA editing factors [114]. Attempts to successfully establish such an in vivo splicing assay focusing on selected mitochondrial group II introns from liverworts, with or without encoded maturases, have remained unsuccessful so far in our laboratory, however (unpublished results). It remains to be seen whether even group II introns in the mitogenomes of early land plants, and possibly even of closely related green algae, rely on yet to be characterized nuclear cofactors [47, 48, 88].

## Materials and methods

### Primary nucleotide sequence data sampling

Streptophyte group II intron sequences were extracted from GenBank accessions available at the National Center for Biotechnology Information (see Table 1 for the primary selection). The sampling also included the recently determined mitogenome of the leptosporangiate fern *Haplopteris ensiformis*, which revealed introns that are not present in the two available mitogenomes of eusporangiate ferns [115]. Introns were labeled using the standard nomenclature proposal indicating the respective gene and the homologous nucleotide position in the *Marchantia polymorpha* mitogenome reference [57, 58]. Introns were manually re-checked for conserved sequence motifs at their terminal 5′- and 3′-splicing sites and corrections were applied where necessary (see examples under results). Mitogenome accessions were also checked for potentially overlooked group II introns by taking advantage of searching for their characteristic and mostly conserved domains V of 34 bp [59]. Individual introns were used as queries in sensitive BLASTN searches to identify significant similarities also in intergenic sequences (IGS) of mitogenomes to identify cases of “intron fossils” (see main text). Sensitive BLASTN search parameters were set as follows: word size of 7, match and mismatch values of 2 and -3 and penalty values of 5 and 2 for gap opening and extension and a random expectancy cutoff value of 1e-5, respectively.

**Table 1:** List of Species-data used for different analyses

Major lineage		Species	Acc. Number	Mt- group II introns
**Streptophyte algae**	Coleochaetophyceae	*Chaetosphaeridium globosum*	NC_004118.1	2 +
	Coleochaetophyceae	*Coleochaete scutata*	NC_045180.1	26 +
	Mesostigmatophyceae	*Mesostigma viride*	AF353999	3
	Chlorokybophyceae	*Chlorokybus atmophyticus*	EF463011.1	14 +
	Charophyceae	*Nitella hyalina*	NC_017598	8
	Charophyceae	*Chara vulgaris*	NC_005255.1	13 +
	Klebsormidiophyceae	*Klebsormidium flaccidum*	KP165386	4
	Zygnematophyceae	*Closterium baillyanum*	NC_022860.1	12 +
	Zygnematophyceae	*Roya obtusa*	NC_22863.1	2
	Zygnematophyceae	*Entransia fimbriata*	NC_22861.1	2
	Zygnematophyceae	*Zygnema circumcarinatum*	MT040698.1	10 +
	Zygnematophyceae	*Gonatozygon brebissonii*	NC_046951.1	6
**Liverworts**	Haplomitriopsida	*Treubia lacunosa*	NC_016122.1	21 +
	Marchantiopsida	*Marchantia polymorpha*	MK202951.1	25 +
	Jungermanniopsida	*Calypogeia fissa*	NC_035980	19 +
	Jungermanniopsida	*Pleurozia purpurea*	NC_013444	22
**Mosses**	Sphagnopsida	Flatbergium novo-caledoniae*	KU725492	26 +
	Sphagnopsida	*Sphagnum palustre*	NC_024521.1	26 +
	Bryopsida	*Physcomitrium patens*	NC_007945.1	24 +
	Bryopsida	*Anomodon rugelii*	NC_016121	24
	Bryopsida	*Ulota crispa*	NC_031393	24
**Hornworts**	Leiosporocerotopsida	*Leiosporoceros dussii*	NC_039751.1	34 +
	Anthocerotopsida	*Anthoceros agrestis*	NC_049004.1	39 +
	Anthocerotopsida	*Nothoceros aenigmaticus*	EU660574	28 +
	Anthocerotopsida	*Phaeoceros laevis*	NC_013765	31
**Lycophytes**	Lycopodiopsida	*Phlegmariurus squarrosus*	NC_017755.1	37 +
	Lycopodiopsida	*Isoetes engelmannii*	FJ390841.1, FJ176330.1, FJ010859.1, FJ628360.1	23
	Lycopodiopsida	*Selaginella moellendorffii*	JF338143.1–JF338147.1	33
**Ferns (Monilophytes)**	Polypodiopsida	*Ophioglossum californicum*	NC_030900.1	20
	Polypodiopsida	*Psilotum nudum*	NC_030952 & KX171639.1	24 +
	Polypodiopsida	*Haplopteris ensiformis*	OM867545- OM867553	24 +
**Gymnosperms**	Ginkgoopsida	*Ginkgo biloba*	NC_027976.1	25 +
	Cycadopsida	*Cycas taitungensis*	NC_010303	25 +
	Gnetopsida	*Welwitschia mirabilis*	NC_029130	10
**Angisoperms**	Magnoliopsida	*Amborella trichopoda*	KF754799-KF754803	24
	Magnoliopsida	*Nelumbo nucifera*	NC_030753	23 +
	Magnoliopsida	*Zea mays*	NC_007982.1	22 +
	Magnoliopsida	*Arabidopsis thaliana*	NC_037304.1	23 +

List of taxa and mitogenomes for detailed initial scoring of mitochondrial group II intron presence. The respective numbers of group II introns are indicated. Taxon selection was aimed to maximize phylogenetic diversity within the respective clades. Care was taken to extract intron sequences with proper 5′- and 3′-termini. Asterisks indicate species not further considered for detailed group II intron sequence analyses owing to redundancy with other species within the respective group. A plus sign (+) in the IGS analysis column indicated careful inspection of intergenic sequences for the presence of intron fossils

### Maturase sequence sampling

Mitochondrial and nuclear encoded maturases were collected independently of the primary group II intron sequences, extending an earlier sampling [113] with sequences from streptophyte algae, also including evident maturases that have not been annotated in sequence entries (see Additional file 4: Fig. S2). The conservation and disintegration of the respective maturases was evaluated with respect to subdomains RT0–RT7, including signature peptide motifs [116] as well as the conservation of the X-domain and signature peptide motifs for DNA-endonucleases [117]. The ultimate set of streptophyte mitochondrial maturases was used for BLASTP searches for homologs with significant similarities also outside of the plant mitochondrial lineage in other eukaryotic lineages: Metazoa, Fungi, Stramenopiles, Oomycetes, Cryptophytes, Rhodophytes and Chlorophytes. The top ten significant hits for each query were retained after removal of duplicates for alignment and phylogenetic tree construction (see Additional file 5: Fig. S3).

### The “most-distant ortholog” concept to define core primary group II intron families

Starting from occasional observations that streptophyte mitochondrial group II intron paralogs in different insertion sites may share more sequence similarities than orthologs in phylogenetically distant clades, we developed a “most-distant-ortholog”-concept (MDO) to cluster the collection of 161 introns (781 representatives in the initial collection of organelle genomes, see Table 1) into families of related paralogs. The full collection of group II intron sequences was initially compared against itself using the stand-alone BLAST + tool [118]. Results were evaluated using the Rstudio package “dplyr” of the “tidyverse library” [119]. Initial clustering following the MDO criterion used the BLASTN search bit score values for most distant orthologs as cut-off to consider paralog hits with higher bit scores for inclusion into a given family.

### Refinement of the group II intron family concepts

Intron homologs present at evidently identical insertion sites that failed to be included in the initial orthologous clustering (“false negatives”) owing to large, “unbridged” phylogenetic distances likely resulting from (multiple) losses (e.g., rps3i74g2 present in charophyceaen algae, hornworts and tracheophytes) were included among the respective family sampling if not showing higher similarity to members of another family. Taxonomically isolated introns receiving only low bit scores of less than 200 were manually re-inspected and only considered for family inclusion when independent sequence similarities were identified in multiple intron regions and/or clearly responsible for conserved RNA secondary structures (see next paragraph). Vice versa, we wished to exclude false positive family assignments for paralogs with arbitrarily high bit scores owing to taxon-specific sequence evolution e.g., due to independently arising repeat motifs.

### Group II intron alignments, phylogenetic analyses and RNA structure modeling

The clustering of intron orthologs and related paralogs into families was manually re-evaluated and re-checked by careful inspection of the respective full alignments. Intron sequences were automatically aligned with MAFFT [120] followed by manual adjustment where necessary. Sequence conservation was visualized with GeneDoc 2.7 (https://genedoc.software.informer.com/). Alignment positions with 60% site coverage identified in MEGA 7.0 [121] were extracted for further phylogenetic analyses and modelling of RNA-secondary structures. The filtered group II intron family alignments were used for Maximum Likelihood phylogenetic analyses with IQ-TREE 2 [122]. Substitution model testing was done automatically, and node support was evaluated with 1000 ultra-fast bootstrap and SH-aLRT repetitions, respectively. Phylogenetic Trees were visualized and edited with TreeGraph2 [123]. RNA secondary structures were modelled manually following established group II intron structure modeling routines [77] with predictions for substructure generated by the mxfold2 application [124]. Nucleotide positions from group II intron family alignments with 60% site coverage sites were preferably used for folding compared to non-conserved sites. RNA secondary structures were annotated in Dot-Bracket annotation and visualized with VARNA [125].

### Evaluation of streptophyte mitochondrial group II intron presence and absence

Presence and absence of the full collection of streptophyte mitochondrial group II intron paralogues were coded as ‘1’ and ‘0’, respectively. The R4.1.2 packages “dplyr” and “eulerr” were used for evaluation and plotting of group II intron distributions in the major land plant lineages to create Venn/Euler diagrams [126, 127]. Intron distribution data was converted into the FASTA format to model gain and loss scenarios empirically for group II introns for different plant phylogeny scenarios with GLOOME [128]. GLOOME allows gain–loss models weights 8-to-1, 4-to-1, 2-to-1 and 1-to-1. Given the present evidence for significantly more frequent independent group II intron losses rather than gains as recently confirmed e.g. for *cox2* introns cox2i373g2 and coxi691g2 among angiosperms [129], we estimated the best fitting model weight (4–1) empirically for the lowest total number of gain events as close as possible to 161.

## Supplementary Information

**Additional file 1.**

**Additional file 2.**

**Additional file 3.**

**Additional file 4.**

**Additional file 5.**

**Additional file 6.**

**Additional file 7.**

**Additional file 8.**

## Data Availability

The mitochondrial genomes are deposited under the following accession numbers Nucleotide Archive at NCBI (www.ncbi.nlm.nih.gov/nuccore) listed in Table 1: NC_004118.1,NC_045180.1, AF353999, EF463011.1, NC_017598, NC_005255.1, KP165386, NC_022860.1, NC_22863.1, NC_22861.1, MT040698.1, NC_046951.1, NC_016122.1, MK202951.1, NC_035980, NC_013444, KU725492, NC_024521.1, NC_007945.1, NC_016121, NC_031393, NC_039751.1, NC_049004.1, EU660574, NC_013765, NC_017755.1, FJ390841.1, FJ176330.1, FJ010859.1, FJ628360.1, JF338143.1–JF338147.1, NC_030900.1, NC_030952 & KX171639.1, OM867545-OM867553, NC_027976.1, NC_010303, NC_029130, KF754799, KF754803, NC_030753, NC_007982.1, NC_037304.1.
