# Supplementary material for: Categorizing 161 plant (streptophyte) mitochondrial group II introns into 29 families of related paralogues finds only limited links between intron mobility and intron-borne maturases
Source: BMC Ecol Evol. 2023 Mar 13;23:5. doi: 10.1186/s12862-023-02108-y (PMC10012718; doi:10.1186/s12862-023-02108-y)
Supplement: Supplementary file 1 — Additional file 1. [file 12862_2023_2108_MOESM1_ESM.docx]

Supplementary Material S1

#### Family F18

#### Family F18 contains two intron paralogues presently only identified in algae of the Zygnematophyceae ([Fig. 4](#_Figure_3_–)): nad5i537g2 is present exclusively in Zygnema circumcarinatum and nad7i777g2 is present in Z. circumcarinatum and in Closterium baillyanum, of which only the latter homologue is equipped with an intron-borne maturase ORF.Family F20

Intron family F20 comprises two intron paralogues so far exclusively identified in the mitogenome of the alga *Closterium baillyanum* of the Zygnematophyceae: cox3i641g2 and nad9i275g2 ([Fig. 4](#_Figure_3_–)). Neither of the two intron paralogues carries a maturase reading frame.

#### Family F21

Intron family F21 contains introns presently only found in *Coleochaete scutata:* atp1i66g2, nad5i1725g2, rrnSi435g2 and trnH-GUGi32g2 ([Fig. 4](#_Figure_3_–)). Only the nad5i1725g2 paralogue carries a maturase. The size increase of the *Coleochaete scutata* mitogenome in comparison to that of *Chaetosphaeridium globosum*, also in the Coleochaetales, had in part been ascribed to the presence of 57 vs. only 11 introns [68]. We now find the here defined family F21 particularly interesting, because additional intron homologies are also present in multiple intergenic regions of the *Coleochaete* mitogenome: *trnMf-nad9, mttB-trnL* and *trnV-trnD*. In all three cases, homologies sharply coincide with the intron 5’-end and extend for minimally 600 bp indicating retrotransposition rather than events of DNA recombination at their origins ([Suppl. Fig. 1](#_Supplementary_Figure_1_1) J).

#### Family F22

As in the case of F21, intron family F22 also includes members presently only identified in the mitogenome of *Coleochaete scutata*: atp1i850g2 and nad5i362g2 ([Fig. 4](#_Figure_3_–)). Of these two, only atp1i850g2 carries a maturase reading frame.

#### Family F23

Intron family F23 comprises two intron paralogues that are hitherto likewise only identified in streptophyte algae: nad7i925g2 in *Closterium baillyanum* and rrnLi2032g2 identified in *Coleochaete scutata* and *Nitella hyalina* ([Fig. 4](#_Figure_3_–)). Only the latter carries a maturase reading frame.
