# Supplementary material for: Categorizing 161 plant (streptophyte) mitochondrial group II introns into 29 families of related paralogues finds only limited links between intron mobility and intron-borne maturases
Source: BMC Ecol Evol. 2023 Mar 13;23:5. doi: 10.1186/s12862-023-02108-y (PMC10012718; doi:10.1186/s12862-023-02108-y)
Supplement: Supplementary file 2 — Additional file 2. [file 12862_2023_2108_MOESM2_ESM.docx]

Supplementary Material S2

Many of the core solitary group II introns are presently very restricted in occurrence, most notably many introns presently identified in only one algal genus. This category, for example, also includes the unique *trans*-splicing introns nad3i84g2 and nad3i301g2 in the *nad3* gene in *Mesostigma viride*. Among land plants this includes solitary introns exclusively restricted to mosses (atp1i1127g2 and cox1i1200g2), to hornworts (atp1i805g2, atp1i1019g2, cobi838g2, cox1i1298g2, cox2i281g2 and cox3i109g2) and to lycophytes (cobi693g2, cox1i227g2, cox1i266g2 and cox1i995g2), respectively, whereas no solitary introns are identified that are restricted to either ferns, gymnosperms or angiosperms.

Other solitary introns, however, are shared between at least two major plant clades ([Fig. 5](#_Figure_4._Group_1)) e.g., nad4Li283g2 in liverworts and mosses or nad3i52g2 in hornworts and lycophytes. Yet others are shared among all euphyllophytes (nad5i1872g2), all tracheophytes (nad1i394g2, nad2i542g2 and nad7i917g2) or tracheophytes and at least one bryophyte clade (cox2i691g2, nad2i709g2 and nad7i140g2). An interesting further case is trnN-GUUi38g2 which is present in three algal classes likely close to the land plant lineage, absent in liverworts but clearly recognizable in pseudogenized form in mosses, hornworts and *Phlegmariurus* among the lycophytes (Suppl. Fig. 3 N).
