# Supplementary figures and images for: Categorizing 161 plant (streptophyte) mitochondrial group II introns into 29 families of related paralogues finds only limited links between intron mobility and intron-borne maturases
Source: BMC Ecol Evol. 2023 Mar 13;23:5. doi: 10.1186/s12862-023-02108-y (PMC10012718; doi:10.1186/s12862-023-02108-y)

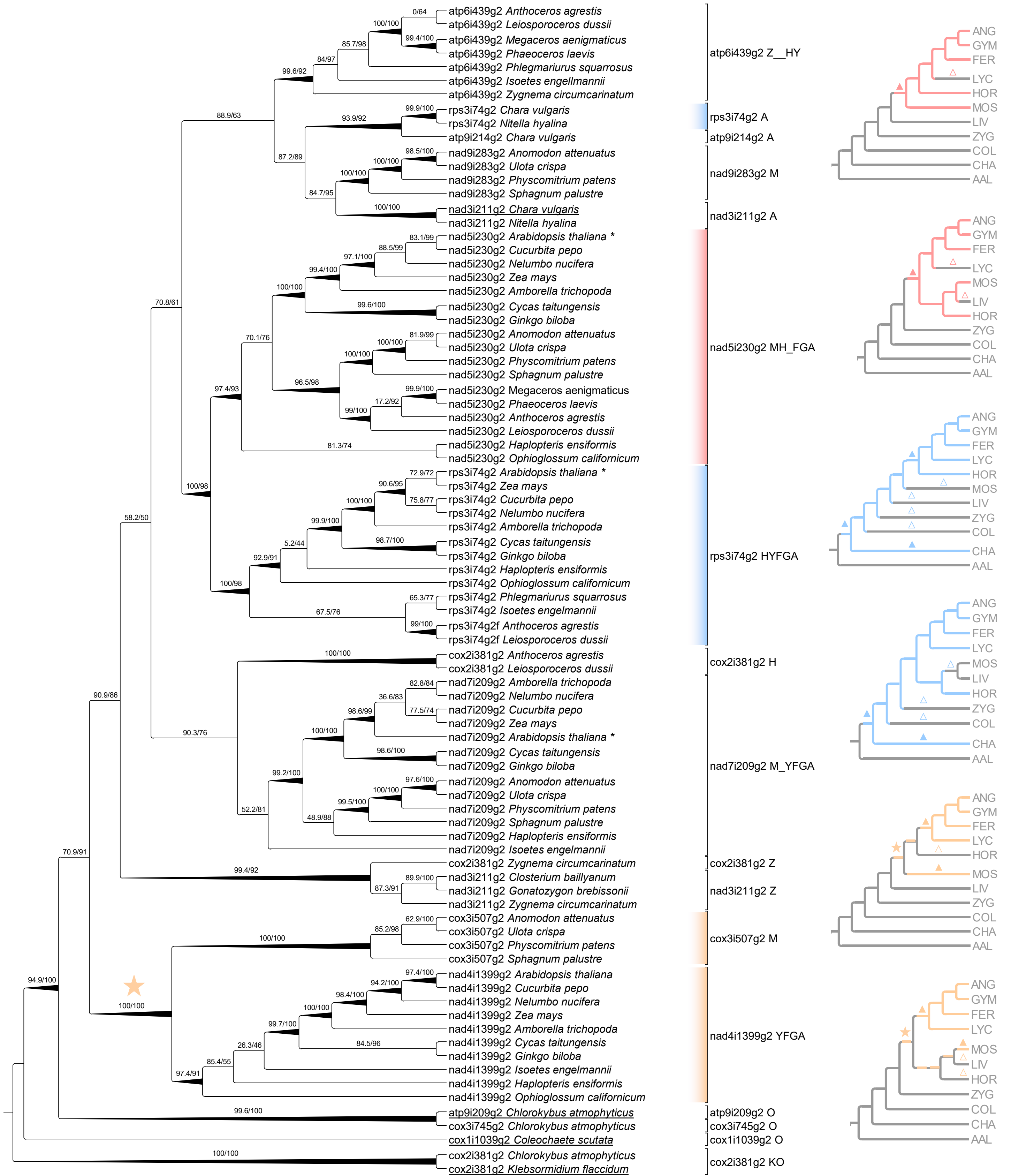

Supplement: Supplementary file 3 — Additional file 3. [file 12862_2023_2108_MOESM3_ESM.pdf]
