# Supplementary material for: Categorizing 161 plant (streptophyte) mitochondrial group II introns into 29 families of related paralogues finds only limited links between intron mobility and intron-borne maturases
Source: BMC Ecol Evol. 2023 Mar 13;23:5. doi: 10.1186/s12862-023-02108-y (PMC10012718; doi:10.1186/s12862-023-02108-y)
Supplement: Supplementary file 4 — Additional file 4. [file 12862_2023_2108_MOESM4_ESM.pdf]

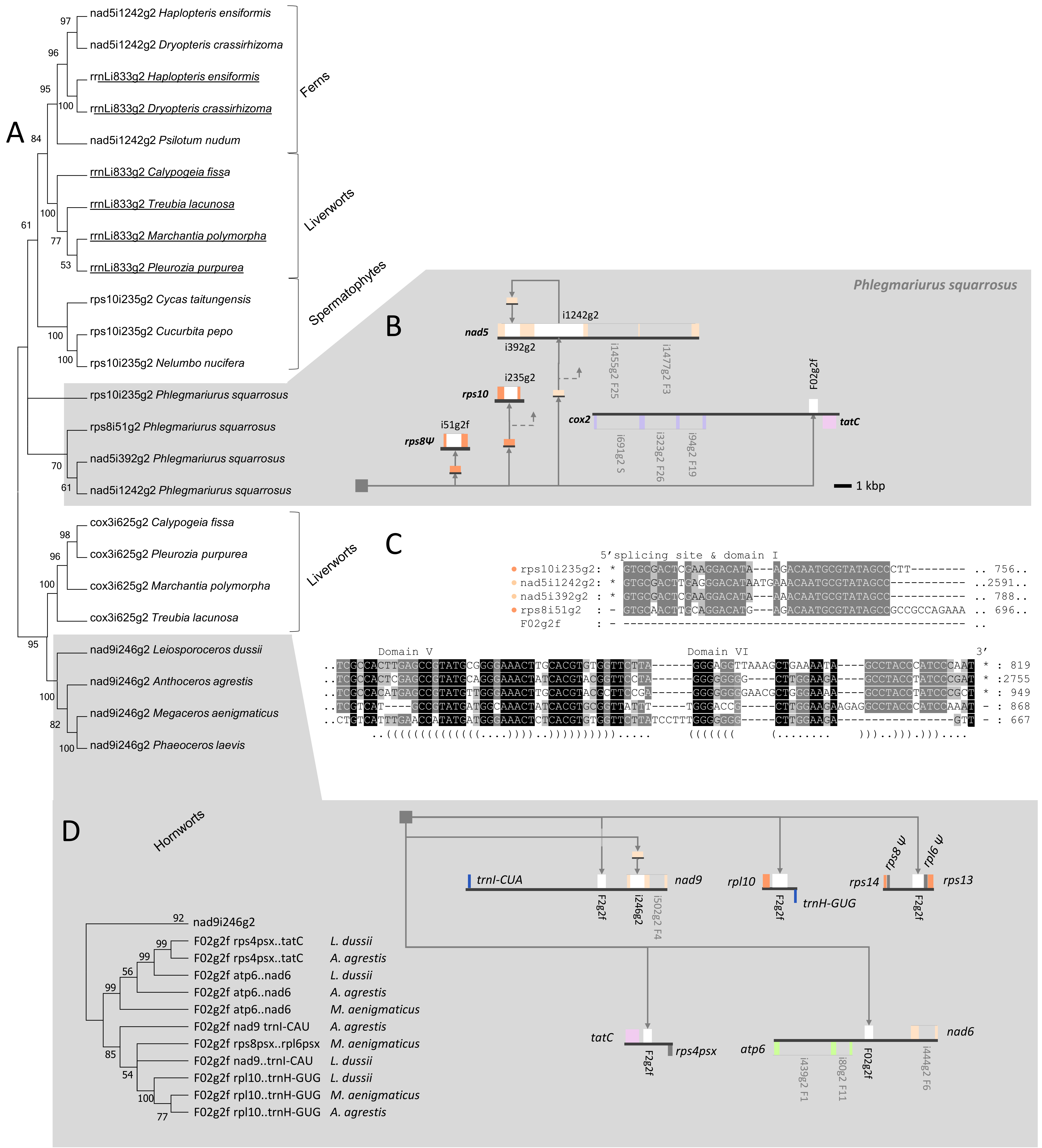

A
nad5i1242g2 Haplopteris ensiformis
nad5i1242g2 Dryopteris crassirhizoma
rrnLi833g2 Haplopteris ensiformis
rrnLi833g2 Dryopteris crassirhizoma
nad5i1242g2 Psilotum nudum
rrnLi833g2 Calypogeia fissa
rrnLi833g2 Treubia lacunosa
rrnLi833g2 Marchantia polymorpha
rrnLi833g2 Pleurozia purpurea
rps10i235g2 Cycas taitungensis
rps10i235g2 Cucurbita pepo
rps10i235g2 Nelumbo nucifera
rps10i235g2 Phlegmariurus squarrosus
rps8i51g2 Phlegmariurus squarrosus
nad5i392g2 Phlegmariurus squarrosus
nad5i1242g2 Phlegmariurus squarrosus
cox3i625g2 Calypogeia fissa
cox3i625g2 Pleurozia purpurea
cox3i625g2 Marchantia polymorpha
cox3i625g2 Treubia lacunosa
nad9i246g2 Leiosporoceros dussii
nad9i246g2 Anthoceros agrestis
nad9i246g2 Megaceros aenigmaticus
nad9i246g2 Phaeoceros laevis
Ferns
Liverworts
Spermatophytes
Liverworts
97 96 95 100 84 100 77 53 61 100 100 70 61 98 96 100 95 100 82 100
B
Phlegmariurus squarrosus
nad5
i1242g2
i392g2
i1455g2 F25
i1477g2 F3
rps10
i235g2
rps8Ψ
i51g2f
cox2
i691g2 S
i323g2 F26
i94g2 F19
F02g2f
tatC
1 kbp
C
5'splicing site & domain I
rps10i235g2: * GTGCGACTCGAAGGACATA---AGACAATGCGTATAGCCCTT--------- .. 756..
nad5i1242g2: * GTGCGACTTCAGGGACATAATGAAACAATGCGTATAGCC----------- ..2591..
nad5i392g2 : * GTGCGACTCGAAGGACATA---AAACAATGCGTATAGCC----------- .. 788..
rps8i51g2 : - GTGCAACTTGCAGGACATG---AGACAATGCGTATAGCCGCCGCCAGAAA .. 696..
F02g2f : - -- ..
Domain V
Domain VI
3'
* : 819
* :2755
* : 949
- : 868
- : 667
D
Hornworts
trnI-CUA
nad9
F2g2f
i246g2
i502g2 F4
rpl10
F2g2f
trnH-GUG
rps14
rps8 Ψ
rpl6 Ψ
rps13
F2g2f
tatC
F2g2f
rps4psx
atp6
i439g2 F1
i80g2 F11
F02g2f
i444g2 F6
nad6
92 nad9i246g2
F02g2f rps4psx..tatC L. dussii
F02g2f rps4psx..tatC A. agrestis
F02g2f atp6..nad6 L. dussii
F02g2f atp6..nad6 A. agrestis
F02g2f atp6..nad6 M. aenigmaticus
F02g2f nad9 trnI-CAU A. agrestis
F02g2f rps8psx..rpl6psx M. aenigmaticus
F02g2f nad9..trnI-CAU L. dussii
F02g2f rpl10..trnH-GUG L. dussii
F02g2f rpl10..trnH-GUG M. aenigmaticus
F02g2f rpl10..trnH-GUG A. agrestis
99 99 56 99 85 54 100 77

Supplementary figure 2 Family F02 of streptophyte mitochondrial group II introns.

A. Detailed phylogeny of group II intron paralogues in family F02. Phylogenetic resolution for F02 paralogues is lower than in the case of F01 introns (Suppl. Fig. 16). For simplicity, only a simple ML consensus tree for nodes >50% obtained with MEGA7.0 is shown. Paralogues cox3i625g2 in liverworts and nad9i246g2 in hornworts and introns nad5i1242g2 and rrnLi833g2 in ferns, respectively, emerge as closely related paralogues B. Presence of nad5i1242g2 also in ferns and of rps10i235g2 also in seed plants leaves the series of intron copying events including creation of two intron fossils in *rps8* and in the *tatC-cox2* spacer unclear except for the likely late emergence of nad5i392g2 from nad5i1242g2 only in Lycopodiales. C. Both F02 intron fossils in Phlegmariurus squarrosus have characteristic sequence conservation at the 3' intron end including terminal domains V and VI (indicated by dot-bracket annotation) compared to the functional paralogs, but only rps8i52g2f features additional sequence conservation also at the 5' intron end. D. A F02 group II intron paralog, likely related to nad9i246g2, created multiple intron fossils (F02g2f) in several intergenic regions in hornwort mitogenomes. These intron fossils are widely conserved within the intergenic regions *rpl10..trnH-GUG* and *atp6..nad6* but more restricted for another fossil between pseudogenes for *rps8* and *rpl6* in *Megaceros aenigmaticus*. Altogether five intergenic F02 intron fossils could be discovered in the hornwort mitogenomes, likely originating from nad9i246g2 or a common ancestor. In this case, the two intergenic intron fossils between *rps4* and *tatC* and between *atp6* and *nad6* appear more closely related to each other than the remaining three. The extension of the intron sequence similarities ending with the respective 5'- or 3'-intron termini and their identification in the conservatively evolving mitogenomes of early land plants devoid of heavy recombination as in seed plant mitogenomes strongly argues for their origin by retrotransposition rather than mtDNA recombination.
