# Supplementary material for: Categorizing 161 plant (streptophyte) mitochondrial group II introns into 29 families of related paralogues finds only limited links between intron mobility and intron-borne maturases
Source: BMC Ecol Evol. 2023 Mar 13;23:5. doi: 10.1186/s12862-023-02108-y (PMC10012718; doi:10.1186/s12862-023-02108-y)
Supplement: Supplementary file 5 — Additional file 5. [file 12862_2023_2108_MOESM5_ESM.pdf]

Supplementary Figure 3 – Fossilized group II introns in plant mitochondrial intergenic sequences.

Homologies of plant mitochondrial group II introns in intergenic regions that are sharply terminating with 5’ and/or 3’ intron ends and free of flanking exon homologies are likely examples for intron retro-transpositions rather than resulting from DNA recombination. Such examples were identified for group II intron families F02 (A and B), F03 (C), F06 (D and E), F10 (F), F14 (G), F17 (H), F19 (I), F21 (J), F18 (O) for pseudogene remnants of trnS-GCUi43g2 in F24 (K), and for solitary introns atp1i989g2 (L), atp1i1050g2 (M), and trnN-GUUi38g2 (N).

A

```
Anthoceros agrestis F02g2| Anthoceros agrestis. mtDNA IGS
                        IBS        5’ splicing site
nad9i246g2        1     CTATCCCCCC GTGCGACTTGAGGGACATAAGACCATGC---TATAGCCCTAG---GGCTATAGGTTTA  63
                                || |||| || ||| ||| |    |||||||   ||||| |||||   ||||||||||| |
trnI-CAU 7128bp < 67799 TAGTCTTGCC GTGCCACCTGAAGGATA----ACCATGCGTGTATAGTCCTAGTAGGGCTATAGGTTCA  67854

                     … 80% identity (613/768)
                                                              domain V
                                                               ((((((
               689   CGACCTCGGTTTGCCTGGGCCTAAACTCTTCTTCGACACTGGAGTCGT  736 + 36bp to 3’ splicing site
                     ||||||||| ||||||||||||||||||||||||| |||| ||| |||
               68499 CGACCTCGGGTTGCCTGGGCCTAAACTCTTCTTCGCCACTCGAGGCGT  68546 > 1456bp nad9
```

```
                    IBS        5’ splicing site
nad9i246g2   1      CTATCCCCCC GTGCGACTTGAGGGACATAAGACCATGCGACCATGCTATAGCCC---------TAGGGCTATAGGTTT 52
                               | ||||||| | |               ||||||||||||||||         ||||||||||   |
atp6 368bp < 178579 TGATTCTTTG GCGCGACTTTAAGAAC------------GACCATGCTATAGCCCGCCCTAGGGTAGGGCTATATAGTC 178489

                  … 78% identity (614/789)
                  domain V
                  ((((((((((.(((....)))...))))))))))                            3’ splicing site
           731    AGTCGTATGCGGGGAAGACTCGCACGTGCGGTTCTCAAGGGAGGGAAGCTTGAGCTTACCTATCCCAATTATCCCAAT TGA 810
                  || ||||||| ||||||||||||     || || || || ||||||||||||||| | ||| || |||| | ||| |   ||
           177874 AGGCGTATGC-GGGAAGACTCGC-----CGCTTTTCCAGCGAGGGAAGCTTGAGCCTCCCTCTCTCAATCAACCCTAA AGA 17782
> 2209bp nad6
```

```
                       IBS        5’ splicing site
nad9i246g2      1      CTATCCCCCC GTGCGACTTGAGGGACATAAGACCATGC-TATAGCCCTAGGGCTATAGGTTTATCCTAGC 70
                        ||| |   | ||  ||||||| ||||    |||||||| | ||||| ||||| |||| |   || | |||
rpl10 55bp <    215693 TTATACGAAC GTATGACTTGAAGGAC----GACCATGCGTCTAGCC-TAGGG-TATATGGCCATTCCAGC 215626

                       … 71% identity (540/765)
                                  domain V
                                  ((((((((((.(((....)))...))))))))))
                719    CTTCGACACTGGAGTCGTATGCGGGGAAGACTCGCACGTGCGGTT  763 > 36bp to 3’ spl. site
                       |||||||||| ||| ||||||  ||||| |||   ||||||| ||
                215006 CTTCGACACTAGAGGCGTATGATGGGAA-ACTATAACGTGCGTTT  214963 > 437bp trnH-GUG
```

```
                         IBS        5’ splicing site
nad9i246g2         1     CTATCCCCCC GTGCGACTTG---AGGGACATAAGACCATGCTATAGCCCTAGGGCT-----ATAGG 48
                          ||     |  | |||| |||   ||   ||| ||||||||| ||| | | ||         |  ||
trnS-GCU 2307bp <  51288 TTAGAGGGCT GCGCGATTTGAGAAGAAGCATGAGACCATGCCATACCTCCAGAATCCACAAACGGG 51219

                   49    ---------ATCCTAGCCGACGGGATGTTCCTACCTCACCATGCGCTCCTGGCCACGGAGAGAGTATGA 112
                                  ||||   ||||||||||||||||||| ||| | ||   || |   ||||  |   | |||
                   51215 TTTTTGGCCATCCCGACCGACGGGATGTTCCTACCCCACTAGGCCGGCCCGATAACGGGCAAGATTTGA 51145

                         … 70% identity (427/608)
                         domain V
                         )...))))))))                       3’splicing site
                   751   CGCACGTGCGGTTCTCA----AGGGAGGGAAGCTTGAGCTTACCTATCCC AAT  799
                         |||| |||   |||| |    | ||||| ||||| |||||||||||||||  ||
                   50535 CGCAAGTGTTTTTCTTATCTCAAGGAGGAAAGCTCGAGCTTACCTATCCC GAT  50483
                                                                    *  G  S   L – C-terminus of tatC
```

B

```
Anthoceros agrestis. F02g2| Megaceros aenigmaticus. mtDNA
                          IBS        5’ splicing site
nad9i246g2   1       CTATCCCCC GTGCGACTTGAGGGACATAAGACCATGC-TATAGCCCTAGGGCTATA-GGTTTATCCTAG 69
                      |||    |  ||||||||| |||     | |||||||| |||| |||| |||||||| |||| || | ||
rps8x 68bp < 46828   ATATCAAGCT GTGCGACTTAAGG-----ACGACCATGCGTATAACCCTGGGGCTATAAGGTTCATTCCAG 46762

          … 61% identity (408/661)
                                        domain V
                                        ((((((((((.(((....)))...))))))))))
      687   TTTGCCTGGGCCTAAACTCTTCTTCGACACTGGAGTCGTATGCGGGGAAGACTCGCACGTGCGGTTCTCAAGGGAG 762  > 26bp 3’spl.
            |||||| |||||||||||||||||||||||||||| ||||||||| ||| ||||||||||||| |||| || ||||
      46228 TTTGCCGGGGCCTAAACTCTTCTTCGACACTGGAGGCGTATGCGGAGAAAACTCGCACGTGCGCTTCTAAAAGGAG 46153 > 53bp rpl6x
```

C

```
Phlegmariurus squarrosus. F03g2| Phlegmariurus squarrosus. mtDNA IGS
                          IBS        5’ splicing site
atp9i21g2       1       TGCAAAATTA GCGCGACCCGTTGGTCTACTACAAGCTTGAAAGTGATGGAGGGTGAAAAT  60
                        |||| ||||| |  ||||||||||| |||||||||||||||||| ||| || |||||||
sdh3 2140bp <   363897  TGCAGAATTA GTACGACCCGTTGGCCTACTACAAGCTTGAAAGTAATGAAGAGTGAAAAA  363838

        … 74% identity (948/1273)

1154    TTGTATAATGGACATGTGGCTAGCTTTTTATTTATCCCAACTCGGCCCACTAGGGTAAAGACCACTAGGaaaa 1226   > 388bp 3’ spl.
        |       |||| ||||||||||||||   ||  ||| ||||||||||||| |||||||||||| | | |||
362789  T-------TGGAAATGTGGCTAGCTTT---TTCCTCCAAACTCGGCCCACTTGGGTAAAGACCACAAAGCAAA 362727 > 2037bp trnV-UAC
```

D

```
Marchantia polymorpha F06g2| Marchantia polymorpha mtDNA IGS

                     IBS        5’ splicing site
cobi783g2  1       CAACTATATA GGGCGACCGGTCTAGATCCCGCACGATAAAAAGCACAAGTCCATTTCTGTGCAAAGG 77
                    |  |  ||  |||||||||       ||||||||||||||||||||||||||||||||||||||||
nad4 319bp< 6166   TATTTTCATC GGGCGACCG-------TCCCGCACGATAAAAAGCACAAGTCCATTTCTGTGCAAAGG 6107

                   … 99% identity (781/789)

            746    CGGGTTTGAAGAAGCTCCGAAGAAAGTCAGGTTAGAGAGCCGTGTGATGGGCGACTATCCCGTACGGTT  814 > 75bp 3’ spl.
                   ||||||||||||||||||||||||||||||||||||||||||||||||||||  ||| | ||| |||||
            5427   CGGGTTTGAAGAAGCTCCGAAGAAAGTCAGGTTAGAGAGCCGTGTGATGGGTCACTCT-CGGTTCGGTT  5363 > 211bp nad5


                       IBS        5’ splicing site
cobi783g2     1        CAACTATATA GGGCGACCGGTCTAGATCCCGCACGATAAAAAGCACAAGTCCATTTCTGTGCAAAGGC  68
                               || || |||||||||      | || |||||||| | |||||| ||    ||| | |||||
rpl10 486bp < 183945   TTTAGCGCTA GGCCGACCGGTC------CTGCGCGATAAAA-GTACAAGTTCACGA-TGTACTAAGGC  183894

                       … 73% identity (676/923)
                       Domain V  Domain VI
                       ))))))))..((((((
              808      TACGGTTCGGAGAGCACTTGAGTAGCCCAGATCGGTGAACGGGGTAACCCTGGGGCCGCA  867
                       ||||||||| |||||     |||||||||||||||| | || || || |||  |||||
              183170   TACGGTTCGTAGAGC-----AGTAGCCCAGATCGGTTAGCGAGGGAAACCTAAGGCCGTC  183116

                            3’  Splicing site
                                  )).)))))..
              868      TAGGGTGGATTCTTGACTCTAT CCC  892
                       ||||||||| |||| ||| ||| |
              183115   TAGGGTGGACTCTTTACTTTAT CGG  183095 > 733bp trnQ-UUG
```

E

Marchantia polymorpha F06g2| Sphagnum palustre mtDNA IGS

```
                          IBS        5’ splicing site
cobi783g2          1      CAACTATATA GGGCGACCGGTCTAGATCCCGCACGATAAAAAGCACAAGTCCATTTCTGTGCAAA-GGC  68
                           |       | ||| | ||  | ||| ||| |||||| |||  | ||  |||||  ||||| |||| |||
trnM-CAU 471bp <   49615  AATTGGGGAA GGGTGCCCAATTTAGTTCCTGCACGAAAAATTGTACT-GTCCAAATCTGTACAAAAGGC  49682

       … 70% identity (414/592)
                                                                                3’ Splicing site
       Domain VI
       (((((((                                                                          )).))))..
818    AGAGCACTTGAGTAGCCCAGA------------------TCGGTGAACGGGGTAACCCTGGGGCCGCATAGGGTGGATTCTTGACTCTAT CCC 892
       ||||||| |  |||||| |||                  |  ||||||   | || |||    | |   | ||| || |||| | |||||  |
50542  AGAGCACCTATGTAGCCAAGAGCACCTATGTAGCCTGCCTTAGTGAACAAAGGAAACCTACGACTGGGCA-GGTAGACTCTTTATTCTAT TCA 50616
> 14bp rrn5
```

Marchantia polymorpha F06g2| Anthoceros agrestis mtDNA IGS

```
                       IBS        5’ splicing site
cobi783g2       1      CAACTATATA GGGCGACCGGTCTAGATCCCGCACGATAAAAAGCACA-AGTCCATTTCTGTGCAAA-GGC  68
                            |  |  ||||||||| |  |  ||| |||||| ||| |    | |||| |||  ||| |||| |||
trnM-CAU 59bp < 131698 TGGTAACCTT GGGCGACCGATGGAAGTCCTGCACGAAAAATACGGTATAGTCGATTA-TGTACAAAAGGC  131765

                69     ATTGCACTCATTCTTGTTCGGCAACGAACACGGAGACCTTAGCATGTGCAAGAAGCTCTG  128
                       |||||||| |  |  |  ||  ||| |||||   |||| |||||||||||||||||||||
                131766 ATTGCACTTACCCG-GCGCGATAACAAACACAAGGACCATAGCATGTGCAAGAAGCTCTG  131824

                     … 74% identity (91/123)
```

F

Phlegmariurus squarrosus F10g2| Phlegmariurus squarrosus mtDNA IGS

```
                        IBS        5’ splicing site
cox1i1149g2      1      CATTATGTA GCGCGGGATGTGACCCTGAATAATTCACTGTTGGT-GGAAGCCAATGCCCCGGGACCTTG  59
                        ||      | |||||||| |||||| ||||||||||||||| ||| || ||||  | |   |||||   |
trnY-GUA 788bp < 404513 CAAACGTAA GCGCGGGAGGTGACCGTGAATAATTCACTGTGGGTAGGTAGCCCTTACG--GGGAC---G  404567

                   … identity 76% (945/1232)
                                                                3’ splicing site
                                       Domain VI
                                    ...(((((((((((((..((....))..))).))))))).)))...
           3532    ACGCTCACGTACAGTTCTGTGAGAAGGGCAGCAAACGGAAATGGCTGCTTGCTCTTACTCTCG CTT 3597
                   |    || |||| ||||| ||    | |||||  ||||| ||||| ||||||||||||||| | | |
           405103  A----CATGTACGGTTCTCTGTTTTGAGCAGCGGACGGAGATGGCCGCTTGCTCTTACTCTTG CCT 405164 > 3945bp nad4
```

G

Coleochaete scutata F14g2| Coleochaete scutata mtDNA IGS

```
                       IBS       5’ splicing site
cox1i511g2       1    TCATCACAA GTGCGCCGCGCGAAGCACACATTCGGCATGGGACCTCTACCCAGGCCCATCGTATATGTT  60 .. 288
                       | |      ||| |||| | || |  ||| | |||  || |  ||  ||| |||| ||||||| || ||
cox1  8737 bp < 86011 CCTTTTGTT GTGTGCCGTGTGAGGTTCACGTCCGGTTTGAGCTCTTAACCTAGGCTCATCGTACATATT  86070 >
19,914bp rrnS
```

H

Sphagnum palustre F17g2 | Phlegmariurus squarrosus mtDNA IGS

```
                                           Domain V                    Domain VI
                                          (((((((((((    ))))  ))))))))...
nad4i461g2        612     GCTATCTCAAGATGGAGCCGTATGATAGGAGACTATCACGTACGGTTCTCTGAGAAGGGA  671
                          |||||||| ||| |||||||||||||  |||| || ||||||||||||||||| |||||
trnM-CAU 1619bp < 352542  GCTATCTCGAGACGGAGCCGTATGATGCGAGAGTACCACGTACGGTTCTCTGAGGAGGGA  352482

  672     GTGGGTACCTACAGGAGCCTTTTCTTAACTTACTCATCATGGGGAGATAAAGCA  728
          ||||||||         ||||| |    || || || |  |||||||||||||
  352483  GTGGGTAC---------CCTTTCC----CTGACCCA-CCGGGGGAGATAAAGCG  352442
```

I

Sphagnum palustre F19g2 | Anthoceros agrestis mtDNA IGS

```
cox1i323g2      332     GGAGGGCAGAACTGAAATCGAACGAGACGGAAAGGATGATAACCATAAGGACGCTGGGTT  391
                        |||| ||| ||| ||||||  |||||||| ||||  | ||||  |   ||||||||| ||
nad9 3913bp < 103703    GGAGAGCAAAACCGAAATCAGACGAGACGAAAAGAGTAATAAAGA---GGACGCTGGATT  103759

818/1247 (66%) - 558/819 (68%)

cox1i323g2  2772    TGGGAGACTATCATGTACGGCTTCGAGGGAGGGAAAAAA  2810
                    ||||||||     | || ||||||||||||||||||||
            106212  TGGGAGAC-----TTTATGGCTTCGAGGGAGGGAAAAAA  106245 > 2885bp trnI-CAU
```

J

Coleochaete scutata F21g2 | Coleochaete scutata mtDNA IGS

```
trnH-GUGi38g2     123     CATAACCCACATCTTGAGACTGGCTATCAAGGGATGAAGCTGGGTTAAACGGGGTGAAAG  182
                          |||| |||||||| ||||||||||||| ||||||||||||||||||||||||||  |||
trnMf-CAU 258bp < 223055  CATAGCCCACATCCTGAGACTGGCTATTAAGGGATGAAGCTGGGTTAAACGGGGATAAAA  222996

351/481(73%)

 527     GGATATGCCCACGTATCTCACGGATCTCTTAGAATACGAAAGTCACAACGAAAGCCAAAC  586
          || | ||  |||||||| ||   ||  |||| | |||| ||||  ||||||| | ||||
 222640  AGACACGCTAACGTATCTTACAAGTCCATTAG-AGACGAGAGTCGTAACGAAAACTAAAC  222582 > 259bp nad9


trnH-GUGi38g2    410     AAAATGGAACACAGGAACGGGAAAAACCCTATCTTCTGTGCTTCGCACCCAGGCGCCAGC  469
                         |||||||| || ||||||||| |||||||| ||||||||||||||||||| |||| |
trnV-UAC 372bp < 164987  AAAATGGAGCATAGGAACGGGGAAAACCCTGTATTCTGTGCTTCGCACCCAGGTGCCAAC  164928

137/174(79%)

  529     ATATGCCCACGTATCTCACGGATCTCTTAGAATACGAAAGTCACAACGAAAGCC  582
          ||| ||  || ||||| |     ||  |||| | || | ||||  ||||||||||
  164867  ATACGCAAACCTATCTTATAAGTCCATTAG-AGACAAGAGTCGTAACGAAAGCC  164815 > 531bp trnD-GUC


trnH-GUGi38g2    124     ATAACCCACATCTTGAGACTGGCTATCAAGGGATGAAGCTGGGTTAAACGGGGTGAA---  180
                         ||||||||||||||||||||||||||      | ||||||| ||||||| |||| |
trnL-UAG 535bp < 167220  ATAACCCACATCTTGAGACTGGCTACT----GCTGAAGCTAGGTTAAATGGGGAGCTTCT  167165

202/280(72%)

359     GAGC-CCTCTCATTCC--CTCCGTGCATCGGCGTAGTATA  395
        |||| || |  | |||  ||  ||||||||||| | ||||
166992  GAGCTCCGCAAAATCCTCCTATGTGCATCGGCGCACTATA  166953 > 1567bp mttB
```

K

Grey shading is in accordance with conservation

```
                       trnS-GCU 5’ exon    5’ splicing site i43g2
Chlorokybus   134235 CATTGGTTTGCTAAATCA---GTGCGACGAGAAAAGTTATTTAATGCAATGTGGT
Marchantia    49085  CATTGGTTTGCTAAATCA---GTGCGACAAG--AAACTGTGCAATGTAATGTGGT
Sphagnum      116390 CATTGCTTTGCTAAATTT---ATGCGACAAG--AAGCTGTGCGATGTAATGTAGT
Anthoceros    92685  CaTTggtttgctaaatca---GTGCGACGAG--AAGCTGTGTGATGTAATGTGGT
Phlegmariurus 355379 ------TTCGCCGAAGCC----AATAATAAAAAAGCTTATCTAAGCTTTTTTTTT


                                                       3’splicing site
Chlorokybus     ACGTACGGTTCT----AAGGGGGGGAAAGCTTGAAAAGGCCGACCTATCCCA-AC  136307
Marchantia      ACGTACGGTTCT---TAGGGGGGGGAAAGCTTGAGAGGGCCTACCTATCCCG-AC  50085
Sphagnum        ATGTATGGTT---------TAGAGGGAAGCTTGAGGGGGCCTATTTATCTTA-AA  118065
Anthoceros      -------------------------------------------------------   -
Phlegmariurus   GCGTACGGTTCTAACTAAAAGGGGGATAGCTAGGGAAGGCCTCCCCATCCCCGAC  354330
```

L

Marchantia polymorpha atp1i989g2| Phlegmariurus squarrosus mtDNA IGS

```
                                5’splicing site <
atp1i989g2         1       GTGCGCCCCGACGGGACGTAGTATGATTCAAACAATGGTTGGAGGGGAAGTCGCTGGCAGGTACTACCAAA  71
                           ||||  ||   ||||| |||||| ||||  ||| ||||   ||| | ||| ||| || |||| |  ||| ||
trnC-GCA 930 bp < 293674   GTGCAACCTACCGGGGCGTAGTTTGATCTAAAAAATGAGAGGAAGAGAATTCGATGACAGGCAAGACCGAA  293744

                        501/744(67%) + 363/561(65%)

                         DV    .((((((((((((((....)))))..)))))))))).
3256    ACCCCCGGAATGTTCCAGTAGGAGCCGTATGATGGGAAACCATCACGTACGGTTCGGTGA  3315
                  ||||||| ||||||| |||||||||||||| || ||||||||||| ||||
330033  ----------TGTTCCAATAGGAGCTGTATGATGGGAAACTATTACGTACGGTTCAGTGA  330082 > 568 bp rps11
```

M

Marchantia polymorpha atp1i1050g2| Phlegmariurus squarrosus mtDNA IGS

```
5’splicing site <
atp1i1050g2      73      AGGGGTAGTTGGA-CTTTTCCTACCCCAA---GTAGCAACACCGTAGGCGATCTTAACAG  128
                         ||||||||||||| ||||||||||||||   ||||| |||||| |||| ||||||||||
cox1 1562bp <    170015  AGGGGTAGTTGGAACTTTTCCTACCCCAACAAGTAGCTACACCGCAGGCAATCTTAACAG  169956

                 … 73% identity (1725/2371)

                 2454    TCTCCTTAGCACCTTAGCCCTCAAGCATAAGTCCTCAATTTCCGGGATTATAAGAGGGTA  2513
                         ||||||  ||||| ||||||| |||||||||  ||| |  ||   |||| | | |   ||
                 167494  TCTCCTAGGCACCCTAGCCCTTAAGCATAAGAGCTCGAGCTCGAAGATTCTCAAACAATA  167435

                 2514    CGGTAAAGCCCCGAAAATACGAACAATTAA  2543   > 337bp 3’ spl. site
                         ||||||||||||||||||| ||| || ||
                 167434  TGGTAAAGCCCCGAAAATACAAACTATCAA  167405 > 1813bp trnW-CCA
```

N

Nitella hyalina trnN-GUU with i38g2 | Phlegmariurus squarrosus mtDNA IGS

```
                           trnN-GUU 5’ exon                    i38g2
Query  1                   TCTCTAGTAGCTCAGTGGTTAGAGCAAATGGCTGTTAA GTGCGCCGATTTTGACTTCTTT  60
                           ||| |||||||||||||||| ||||||||||||||| | |||||| ||     |  | ||
Sbjct cox2 3899bp< 271185  TCTTTAGTAGCTCAGTGGTTGGAGCAAATGGCTGTTGA GTGCGCTGA-----ATATATTG  271131


Query  61      GTGGAGGATGTAGTT-TTTAACTACGACATTGTTCAGAACATGTCTAAAACAATACCTTA  119
               ||   ||||  ||||      ||||| |||| |||||| ||||||||||||||||||||
Sbjct  271130  GTACGGGATTGAGTTAGCCTCCTACGTCATTTTTCAGAGCATGTCTAAAACAATACCTTA  271071 > 9264bp tatC
```

Nitella hyalina trnN-GUU with i38g2 | Physcomitrium patens mtDNA IGS

```
               trnN-GUU 5’ exon                    i38g2
Query  3      TCTAGTAGCTCAGTGGTTAGAGCAAATGGCTGTTAA GTGCGCCGATTTTGACTTCTTTGT  62
              ||||| || || | |||||| ||||||| ||||||| |||||| || | | | |  ||  |
Sbjct  61289  TCTAGGAGTTCCGCGGTTAGCGCAAATGACTGTTAA GTGCGCTGAATAT-ATTGATTGAT  61347


Query  63     GGAGGATGTAGTTTTTAACTACGACATTGTTCAGAACATGTCTAAAACAATACCTTACCT  122
                ||||| ||| |||| |||| | ||||||||||| ||||||||||  | ||||||||||
Sbjct  61348  AAAGGATTTAGCTTTTTACTATGGCATTGTTCAGAGCATGTCTAAACGACTACCTTACCT  61407
```

Sphagnum palustre cox1i732g2 | Phlegmariurus mtDNA IGS

```
                        3bp < 5’ splicing site
Query  3                         GCGCGATTGCGCTAATAGAAAAGGTGGTACATTGCCCTTACAGCGCCACCTAGGCGAGCA  62
                                 |||| ||||||| ||| |||| || ||||  | |||||||| ||||| |||||||| |||
Sbjct  trnQ-UUG 2915bp < 113768  GCGCAATTGCGCCAATGGAAAGGGCGGTAGCT-GCCCTTACTGCGCCTCCTAGGCGGGCA  113826


2030/2776(73%)


Query  2526    AGCTCGAAAGTAGACT--ACATAGGTTATAGGCATATTCTGCGAAGAGCCCAGTGCTTTG  2583
                ||| || | | | ||  |  || ||||||||||||||| |||||||||| ||||||| |
Sbjct  116384  TGCTAGAGATTGGGCTGGAGGTA-GTTATAGGCATATTCCGCGAAGAGCCTAGTGCTTCG  116442 > 8900bp rps2
```

O

Closterium baillyanum F28g2 | Coleochaete scutata mtDNA IGS

```
                      5’ splicing site
nad7i777g2  1         GTGCGGGGAGAGAGCCTCGCCATTACGCTAACGTCGAGAAGACGCCGTACCGGATCCATGGGT  63
                      |  ||||||  || || | ||||||||||  | ||| |||||||  || |||||||||||||
nad3 2376bp < 164619  GCTCGGGGAACGACCCCCACCATTACGCTGGCATCGCGAAGACGTTGTGCCGGATCCATGGGC 164557

796/1181(67%)..317/472(67%).. 411/615(67%)
                 Domain V                            Domain VI
                 ((((((((((((.(....).)))..)))))))))...(((((((...
   2872    AAAGGGGAGCCGTATGCGACGAAAGCCGCACGTATGGTTCTGTGAGAGGCGGAGCGAAG-  2930
           ||||||||||||||||||  |||||||||||||| |||| ||| ||||||||  ||||| 
   161274  AAAGGGGAGCCGTATGCGGTGAAAGCCGCACGTACGGTTTTGTTAGAGGCGGGCCGAAGT  161215

                                       3’ splicing site
                            )))).)))
   2931    ATAAAAGGTCGAATCCAGCCTACTCTCG  2958
             || ||||||    |||||||||||||
   161214  GGAACAGGTCGGTCGCAGCCTACTCTCG  161187 > 1437 bp rps10
```
