# Supplementary material for: Categorizing 161 plant (streptophyte) mitochondrial group II introns into 29 families of related paralogues finds only limited links between intron mobility and intron-borne maturases
Source: BMC Ecol Evol. 2023 Mar 13;23:5. doi: 10.1186/s12862-023-02108-y (PMC10012718; doi:10.1186/s12862-023-02108-y)
Supplement: Supplementary file 6 — Additional file 6. [file 12862_2023_2108_MOESM6_ESM.pdf]

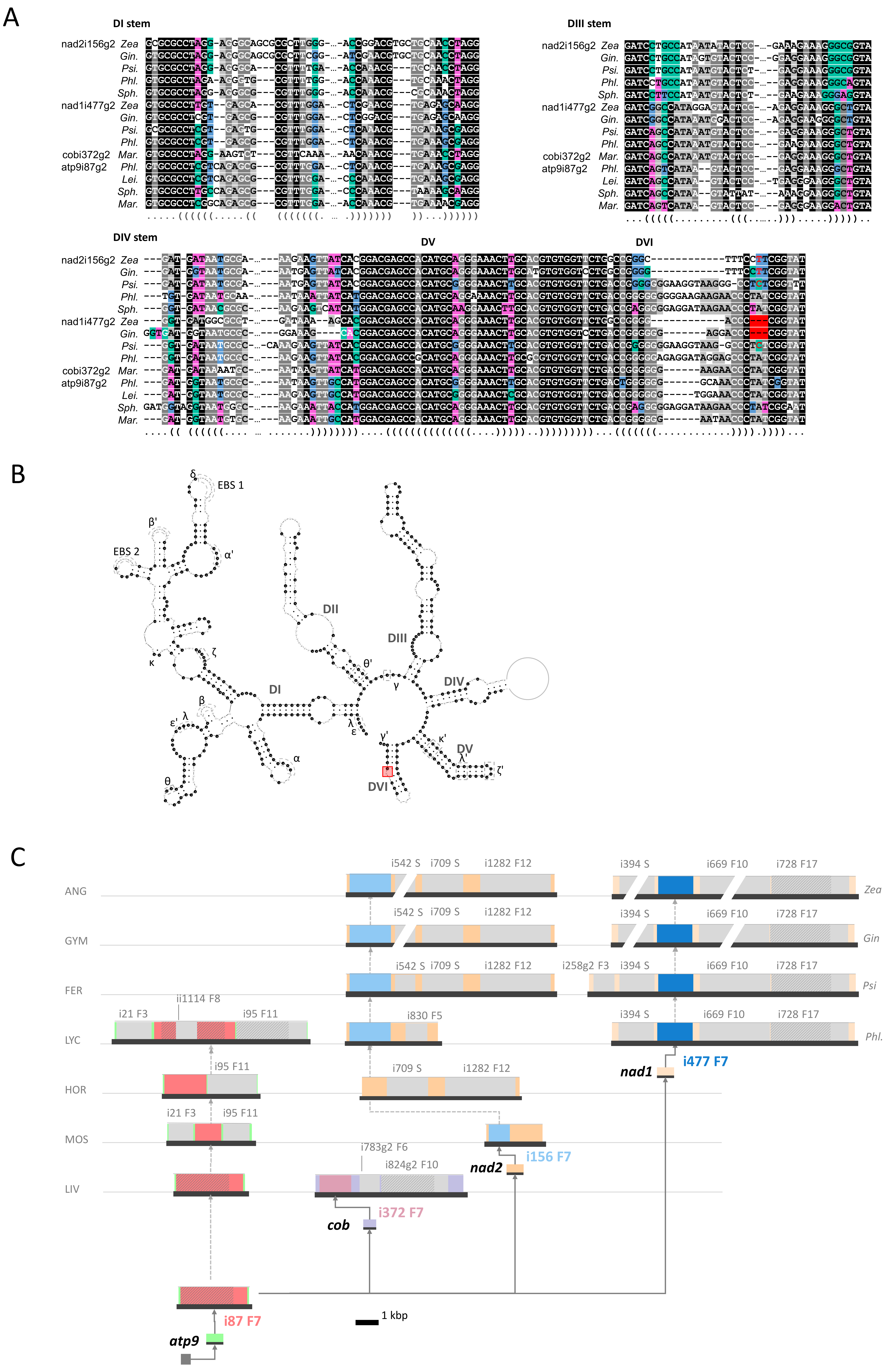

A
DI stem
DIII stem
DIV stem
DV
DVI
nad2i156g2 Zea
Gin.
Psi.
Phl.
Sph.
nad1i477g2 Zea
cobi372g2 Mar.
atp9i87g2 Phl.
Lei.
B
EBS 1
EBS 2
DI
DII
DIII
DIV
DV
DVI
C
ANG
GYM
FER
LYC
HOR
MOS
LIV
Zea
Gin
Psi
Phl.
i542 S
i709 S
i1282 F12
i394 S
i669 F10
i728 F17
i258g2 F3
i830 F5
i21 F3
ii1114 F8
i95 F11
i783g2 F6
i824g2 F10
nad1
i477 F7
nad2
i156 F7
cob
i372 F7
atp9
i87 F7
1 kbp

Supplementary figure 4 Family F07 of streptophyte mitochondrial group II introns.

Group II intron paralogs atp9i87g2, cobi372g2, nad2i156g2 and nad1i477g2 in family F07 feature structurally relevant sequence conservations across the entire intron sequences. A. Exemplary alignments of domain DI and DIII regions and complete domains DV and VI together of selected species *Zea mays* (*Zea*), *Ginkgo biloba* (*Gin*), *Psilotum nudum* (*Psi*), *Phlegmariurus squarrosus* (*Phl*) *Sphagnum palustre* (*Sph*), *Marchantia polymorpha* (*Mar*), *Leiosporoceros dussii* (*Lei*) with dot bracket annotations indicating base pairings. Co-conversions that do affect sequence conservation but not RNA secondary structures are colored based on base-pairing (A-T in pink, G-C in green; G-T in blue). Red boxes and shading indicate degeneration of the conserved bulged A in domain VI, usually involved in lariat formation during splicing reaction. B. Secondary structure of F07-type group II introns based on overall conserved regions of the paralogs. Roman numerals indicate the six typically conserved domains of group II introns (DI-DVI) with EBS 1 & 2 in DI and Greek letters designate tertiary interactions sites, respectively [33]. Black shading of nucleotides shown in panel A corresponds to equally shaded nucleotides in the secondary structure model. C. Intron atp9i87g2 (red box) is the only group II intron conserved in all three bryophyte lineages and additionally present in lycophytes. It encodes maturase mat-atp9i87g2c in liverworts (pattern fill) but this is absent in the moss and hornwort orthologues. In the lycophyte *P. squarrosus* mat-atp9i87g2 is interrupted by internal intron ii1114g2. Ancestral intron atp9i87g2 likely gave rise to cobi372g2 (pink box) in liverworts (LIV), to nad2i156g2 (light blue box) in mosses (MOS) and to nad1i477g2 (blue box) in the tracheophyte lineage, first arising in lycophytes (LYC). Additional group II introns (grey) and their family assignments are indicated. Transitions from cis- to trans-splicing in seed plants are indicated with diagonal interruptions.
