# Supplementary material for: Categorizing 161 plant (streptophyte) mitochondrial group II introns into 29 families of related paralogues finds only limited links between intron mobility and intron-borne maturases
Source: BMC Ecol Evol. 2023 Mar 13;23:5. doi: 10.1186/s12862-023-02108-y (PMC10012718; doi:10.1186/s12862-023-02108-y)

Supplementary Figure 5 – Phylogeny of plant mitochondrial group II intron maturases and related homologues outside of plants.

Phylogram derived from an alignment of maturases sampled by starting from the "seed set" of streptophyte mitochondrial maturases (green, bold). Homologs from evolutionary distant taxa are colored differently: Chlorophytes (CRL) in light green, Rhodophytes (RHD) in red, fungi (FNG) in light brown, Cryptophyceae (CRY) in pink, Metazoa (MET) in light blue, Stramenopiles (SRM) in purple and Oomycetes in black. Nuclear-encoded maturases of the land plant lineage are highlighted in dark green with italics and underlining. Maturase-based intron families (mF) and superfamilies (SF) are indicated. The phylogenetic tree was obtained using IQ-Tree. Model test resulted in the WAG+F+R8 substitution model according to Bayesian Information Criterion. Node support is indicated in percentiles for bootstrap-support and SH-aLRT before and after the slash, respectively. Branches with support above 85|90 are enlarged.

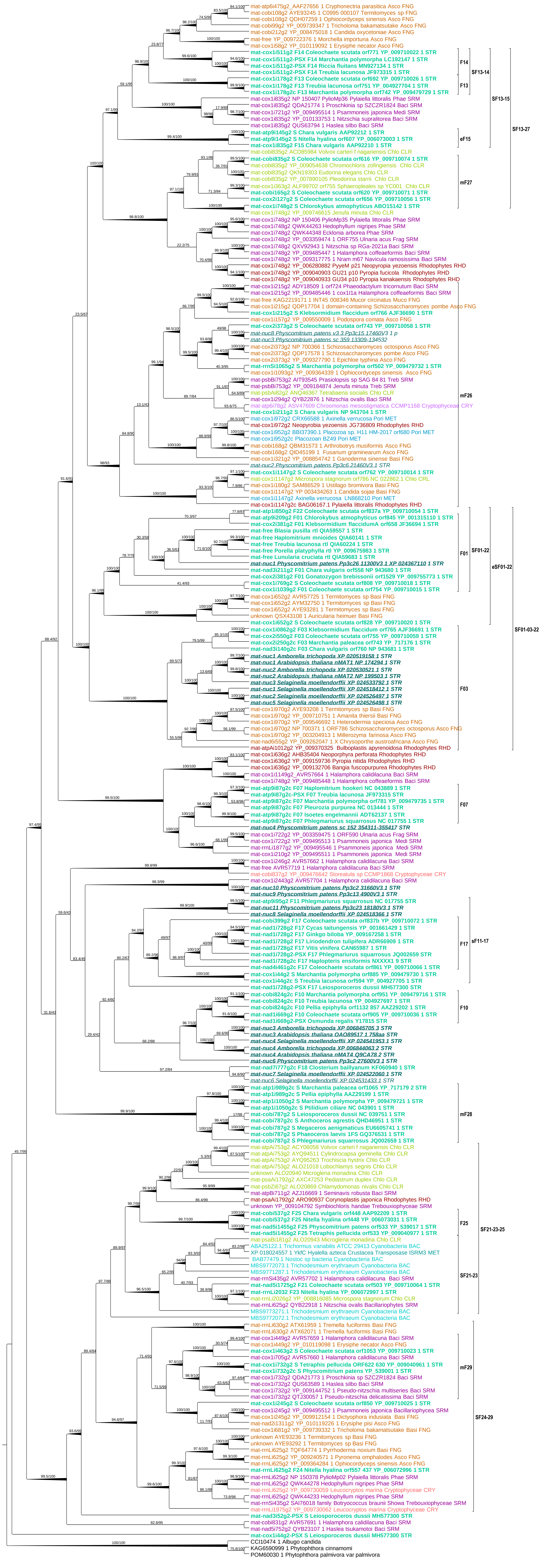

Supplement: Supplementary file 7 — Additional file 7. [file 12862_2023_2108_MOESM7_ESM.pdf]
