# Supplementary material for: Categorizing 161 plant (streptophyte) mitochondrial group II introns into 29 families of related paralogues finds only limited links between intron mobility and intron-borne maturases
Source: BMC Ecol Evol. 2023 Mar 13;23:5. doi: 10.1186/s12862-023-02108-y (PMC10012718; doi:10.1186/s12862-023-02108-y)
Supplement: Supplementary file 8 — Additional file 8. [file 12862_2023_2108_MOESM8_ESM.pdf]

Supplementary Table 1

List of group II intron fossils detected in the intergenic sequences (IGS) in the mtDNAs of the alga *Coleochaete scutata* , the hornwort *Anthoceros agrestis* and the lycophyte *Phlegmariurus squarrosus* .

from	to	size	fossil	IGS	IGS size	closest paralog	identities			
Coleochate scutata **mtDNA NC_045180**									**bp**	**%**
75714	75960	247	F14g2f	*cox1..rrnS*	23902	*C. scutata* cox1i511g2 F14	199/250(80%)	mtDNA	**242024**	
28914	29136	223	F24g2f	*trnMf-CAU..rrnS*	34851	*N. hyalina* rrnLi2032g2 F24	192/230(83%)	g2 introns	**57183**	**23.63%**
36860	37579	720	mF28g2f(1)	*trnMf-CAU..rrnS*	34851	*M. polymorpha* atp1i989g2	200/286(70%)	fossils	**8302**	**3.43%**
39154	39914	761	mF28g2f(2)	*trnMf-CAU..rrnS*	34851	*M. polymorpha* atp1i989g2	190/274(69%)			
138259	138694	436	F18g2f(2)	*rps2..nad6*	3738	*C. baillyanum* nad7i777g2 F18	314/444(71%)			
161187	164616	3430	F18g2f(1)	*nad3..rps10*	7246	*C. baillyanum* nad7i777g2 F18	796/1181(67%);411/615(67%); 317/472(67%)			
174526	175134	609	F01g2f2	*rps12..trnR-UCG*	1604	*C. scutata* cox1i1039g2 F01	77/89(87%); 217/319(68%)			
180324	180949	626	F21g2f(1)	*trnMf-CAU..nad9*	992	*C. scutata* atp1i66g2 F21	438/630(70%)			
188378	188522	145	F18g2f(3)	*atp1..trnN-GUU*	1208	*C. baillyanum* nad7i777g2 F18	113/154(73%)			
203144	203271	128	F24g2f	*atp9..nad5*	2423	*C. scutata* rrnLi629g2 F24	122/128(95%)			
236350	236787	438	F21g2f(2)	*mttB..trnL-UAG*	2214	*C. scutata* nad5i1725g2 F21	331/449(74%)			
238375	238913	539	F21g2f(3)	*trnV-UAC..trnD-GUC*	1076	*C. scutata* nad5i1725g2 F21	431/597(72%)			
Anthoceros agrestis **mtDNA MK087647**									**bp**	**%**
7930	8672	743	F02g2f(2)	*nad6..atp6*	3357	*A. agrestis* nad9i246g2 F02	614/789(78%)	mtDNA	**227925**	
45084	45801	718	F02g2f(3)	*trnH-GUG..rpl10*	1223	*A. agrestis* nad9i246g2 F02	540/765(71%)	g2 introns	**84538**	**37.09%**
47537	48521	985	ccmFci829g2-PSX	*trnF-GAA..trnQ-UUG*	2855	*P. patens* ccmFci829g2 F09	359/469(77%)	fossils	**6185**	**2.71%**
103449	104388	940	sdh3i100g2-PSX	*trnP-UGG..sdh4*	1925	*L. dussii* sdh3i100g2 F03	788/980(80%)			
108669	109112	444	F02g2f(4)	*tatC..rps4psx*	1957	*A. agrestis* nad9i246g2 F02	361/466(77%)			
125931	126679	749	F02g2f(1)	*trnI-CAU..nad9*	9341	*A. agrestis* nad9i246g2 F02	613/768(80%)			
156520	158125	1606	rps3i74g2-PSX	*rpl2..atp9*	12165	*P. squarrosus* rps3i74g2 F01	254/303(84%); 475/710(67%)			
Phlegmariurus squarrosus **mtDNA NC_017755**									**bp**	**%**
25873	26711	839	F11g2f	*trnY-GUA..nad4*	5386	*P. squarrosus* atp9i95g2 F11	819/849(96%)	mtDNA	**413530**	
38884	39124	241	F10g2f(1)	*atp9..trnR-ACG*	10546	*P. squarrosus* sdh3i249g2ii42g2 F10	209/248(84%)	g2 introns	**70155**	**16.96%**
113768	116458	2691	mF29g2f	*trnQ-UUG..trnF-GAA*	14525	*S. palustre* cox1i732g2 mF29	2030/2776(73%)	fossils	**17771**	**4.30%**
161932	162314	383	F03g2f(1)	*trnQ-UUG..trnF-GAA*	14525	*P. squarrosus* nad3i140g2 F03	294/395(74%)			
166678	167325	648	F10g2f(2)	*cox1..trnW-CCA*	6080	*P. squarrosus* sdh3i249g2ii42g2 F10	551/684(81%)			
168191	170015	1825	mF28g2f	*cox1..trnW-CCA*	6080	*M. polymorpha* atp1i1015g2 mF28	1225/1710(72%)			
234113	234735	623	F10g2f(3)	*cox3..trnF-GAA*	5571	*P. squarrosus* cox1i1149g2 F10	517/671(77%)			
243447	246296	2850	cobi693g2f	*rps12..trnE-UUC*	7269	*P. squarrosus* cobi693g2 S	1218/1295(94%)			
246743	247337	595	F10g2f(4)	*rps12..trnE-UUC*	7269	*P. squarrosus* cox1i1149g2 F10	429/547(78%)			
276030	276658	629	F10g2f(5)	*tatC..cox2*	13076	*P. squarrosus* cox1i1149g2 F10	558/668(84%)			
300274	300924	651	F10g2f(6)	*trniM-CAU..trnQ-UUG*	774	*P. squarrosus* cox1i1149g2 F10	500/700(71%)			
303481	304135	655	F10g2f(7)	*trnQ-UUG..atp8*	6204	*P. squarrosus* cox1i1149g2 F10	570/677(84%)			
352374	353683	1310	F17g2f(1)	*trnM-CAU..trnA-UGC*	11152	*P. squarrosus* nad4i461g2 F17	1296/1317(98%)			
362727	363884	1158	F03g2f(2)	*trnV-UAC..sdh3*	5349	*P. squarrosus* atp9i21g2 F03	982/1319(74%)			
366424	367087	664	F10g2f(8)	*trnY-GUA..nad4*	5386	*P. squarrosus* cox1i1149g2 F10	550/697(79%)			
377404	378544	1141	F02g2f(2)	*trnY-GUA..nad4*	5386	*P. squarrosus* nad5i1242g2 F02	981/1196(82%)			
394679	395546	868	rps8i53g2-PSX	*rps14..rpl6*	1689	*P. squarrosus* nad5i392g2 F02	349/442(79%)			
